# Clinical Outcomes of Pneumonia and Other Comorbidities in Children Aged 2-59 Months in Lilongwe, Malawi: Protocol for the Prospective Observational Study “Innovative Treatments in Pneumonia”

**DOI:** 10.2196/13377

**Published:** 2019-07-29

**Authors:** Amy Sarah Ginsburg, Susanne May, Evangelyn Nkwopara, Gwen Ambler, Eric D McCollum, Tisungane Mvalo, Ajib Phiri, Norman Lufesi

**Affiliations:** 1 Save the Children Fairfield, CT United States; 2 Department of Biostatistics University of Washington Seattle, WA United States; 3 PATH Seattle, WA United States; 4 Eudowood Division of Pediatric Respiratory Sciences John Hopkins School of Medicine Baltimore, MD United States; 5 University of North Carolina Project: Lilongwe, Central Region Lilongwe Malawi; 6 Department of Paediatrics and Child Health College of Medicine University of Malawi Blantyre Malawi; 7 Ministry of Health, Republic of Malawi Lilongwe Malawi

**Keywords:** childhood pneumonia, comorbidities, outcomes, Africa

## Abstract

**Background:**

Pneumonia is the leading infectious cause of death worldwide among children below 5 years of age. Clinical trials are conducted to determine optimal treatment; however, these trials often exclude children with comorbidities and severe illness.

**Conclusions:**

Given the paucity of data from Africa, African-based research is necessary to establish optimal management of childhood pneumonia in malaria-endemic settings in the region. An expanded evidence base that includes children with pneumonia and other comorbidities, who are at high risk for mortality or have other complications and are therefore typically excluded from childhood pneumonia clinical trials, can contribute to future iterations of the World Health Organization Integrated Management of Childhood Illness guidelines.

**Methods:**

The study enrolled 1000 children with pneumonia presenting to the outpatient departments of Kamuzu Central or Bwaila District Hospitals in Lilongwe, Malawi, who were excluded from concurrent randomized controlled clinical trials investigating fast breathing and chest indrawing pneumonia and who met the inclusion criteria for this prospective observational study. Each child received standard care for their illnesses per Malawian guidelines and hospital protocol and was prospectively followed up with scheduled study visits on days 1, 2 (if hospitalized), 6, 14 (in person), and 30 (by phone). Our primary objectives are to describe the clinical outcomes of children who meet the inclusion criteria for this study and to investigate whether the percentages of children cured at day 14 among those with either fast breathing or chest indrawing pneumonia and comorbidities such as severe malaria, anemia, severe acute malnutrition, or HIV are lower than those in children without these comorbidities in the standard care groups in concurrent clinical trials. This study was approved by the Western Institutional Review Board, Malawi College of Medicine Research and Ethics Committee, and the Malawi Pharmacy, Medicines and Poisons Board.

**Objective:**

This prospective observational study aimed to assess the clinical outcomes of children aged 2-59 months with both pneumonia and other comorbidities in a malaria-endemic region of Malawi.

**Results:**

The Innovative Treatments in Pneumonia project was funded by the Bill and Melinda Gates Foundation (OPP1105080) in April 2014. Enrollment in this study began in 2016, and the primary results are expected in 2019.

**International Registered Report Identifier (IRRID):**

DERR1-10.2196/13377

## Introduction

Pneumonia is the leading infectious cause of childhood mortality worldwide. As part of the Innovative Treatments in Pneumonia (ITIP) project conducted in Lilongwe, Malawi, two randomized controlled clinical trials are evaluating the optimal duration of treatment with amoxicillin for fast breathing (ITIP1) and chest indrawing (ITIP2) childhood pneumonia [1]. Clinical trials evaluating the treatment for pneumonia often exclude children with comorbidities who are at high risk for mortality or have other complications. Pneumonia with comorbidities is common, and many factors determine whether contact with an etiologic agent will result in a severe episode of pneumonia and whether the episode will be fatal [2-6]. These factors can be related to the child (eg, age, sex, and underlying diseases), disease (eg, type of infection), environment, family and its socioeconomic status, or health system and type of care [7]. A systematic review and meta-analysis of risk factors for mortality from acute lower respiratory infections in children under the age of 5 years in low- and middle-income countries found that chronic underlying diseases (odds ratio [OR] 4.76, 95% CI 3.27-6.93), HIV/AIDS (OR 4.68, 95% CI 3.72-5.90), and severe malnutrition (OR 4.27, 95% CI 3.47-5.25) were associated with mortality due to acute lower respiratory infections [7]. In an effort to generate data on pneumonia treatment outcomes among high-risk African children after introduction of the *Haemophilus influenzae* type b and *Streptococcus pneumoniae* conjugate vaccines and to better interpret the results of the concurrent clinical trials, a prospective observational study (ITIP3) to assess the clinical outcomes of children aged 2-59 months with both pneumonia and other comorbidities was conducted. Given the paucity of data from Africa, African-based research is necessary to establish optimal treatment regimens for childhood pneumonia in the region.

## Methods

### Ethical Approval

The study was approved by the Western Institutional Review Board in the state of Washington, USA; the Malawi College of Medicine Research and Ethics Committee, Blantyre, Malawi; and the Malawi Pharmacy, Medicines and Poisons Board. Written informed consent was obtained by trained study staff from all eligible children’s caregivers prior to enrollment.

### Study Design and Settings

**Figure 1:**
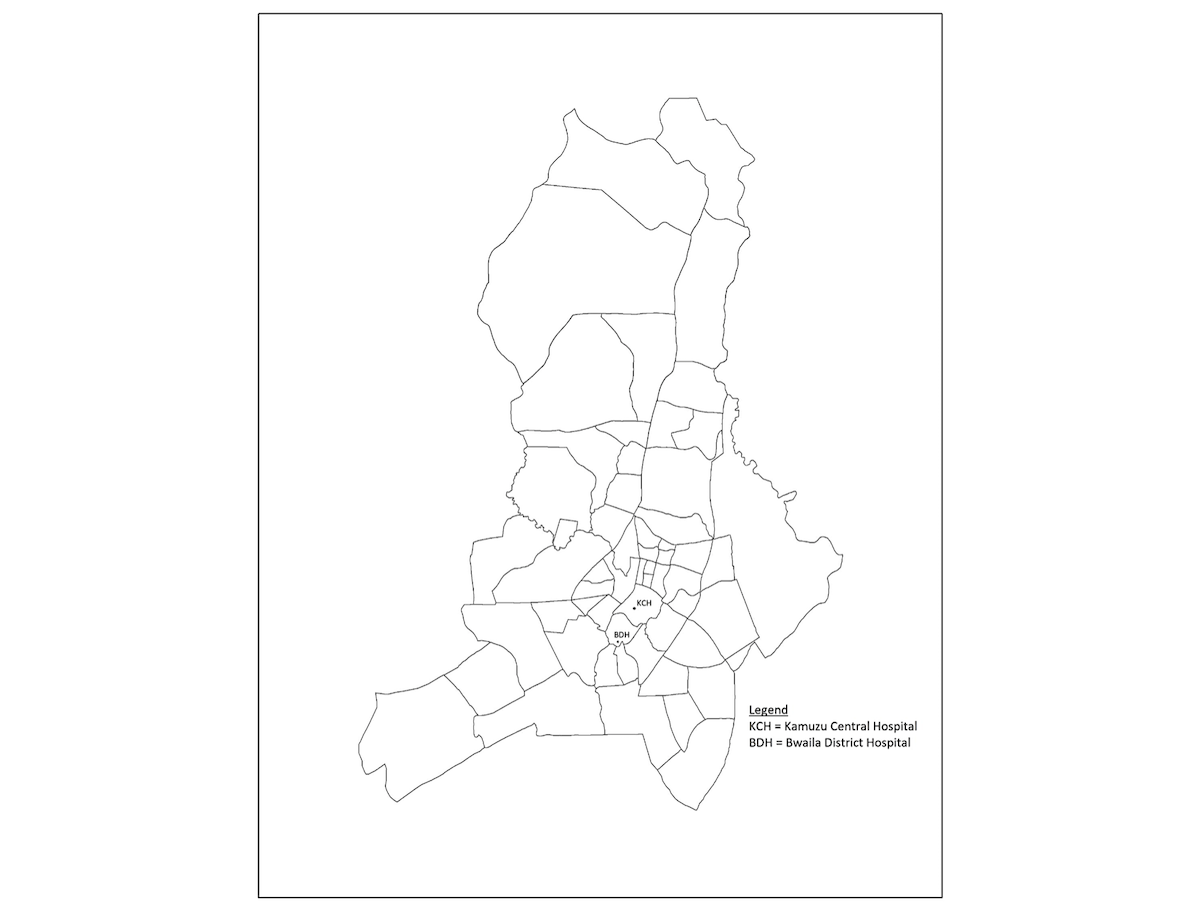
Map of the study area in Lilongwe, Malawi. KCH: Kamuzu; BDH: Bwaila District Hospital.

The primary objective of this prospective, observational study is to determine the clinical outcomes of children aged 2-59 months with pneumonia and other comorbidities in Lilongwe, Malawi, who were excluded from ITIP1 and ITIP2 pneumonia treatment clinical trials and meet the inclusion criteria for ITIP3. We will also investigate whether the percentages of children cured on day 14 from diagnosis among those with either fast breathing or chest indrawing pneumonia and comorbidities such as severe malaria, anemia, severe acute malnutrition, or HIV are lower than those without these comorbidities in the standard care groups in the concurrent clinical trials. The primary aim of the ITIP1 and ITIP2 clinical trials is to provide evidence for the optimal duration of treatment of children with fast breathing or chest indrawing childhood pneumonia (but without other major comorbidities) with amoxicillin dispersible tablets. These two randomized controlled clinical trials are conducted with immunocompetent children aged 2-59 months residing in a malaria-endemic region of Malawi. Children enrolled in the clinical trials are followed for 14 days, with ITIP1 follow-up assessments conducted on days 2, 3, 4, and 14 and ITIP2 follow-up assessments conducted on days 2, 4, 6, and 14. The prospective observational study ITIP3 enrolls children with pneumonia who are excluded from the two clinical trials because of other comorbidities in an effort to provide additional valuable evidence on standard care and outcomes for children with pneumonia in Malawi who are most at risk for mortality or have other complications. An observational design was chosen to follow the clinical outcomes of high-risk children with pneumonia who are typically excluded from clinical trials. This study is conducted at the outpatient and inpatient departments of Kamuzu Central Hospital (KCH) and the outpatient department of Bwaila District Hospital (BDH) in Lilongwe, Malawi (Figure 1). The 750-bed government facility KCH is the primary referral hospital for the central region of Malawi, serving a population of approximately 5 million people. BDH is the district hospital for Lilongwe with no inpatient facilities for children. Children requiring inpatient care are referred to KCH.

### Study Participants

**Figure 2:**
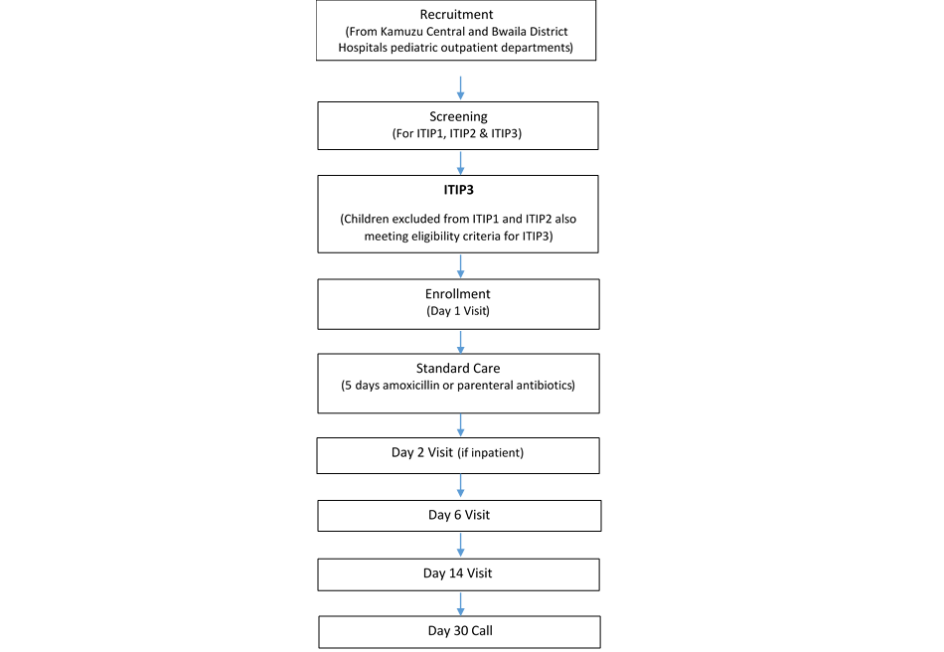
Schedule of enrollment and follow-up assessment. ITIP1: Innovative Treatments in Pneumonia 1, randomized controlled clinical trial evaluating the optimal duration of treatment with amoxicillin for fast breathing pneumonia;ITIP2: Innovative Treatments in Pneumonia 2, randomized controlled clinical trial evaluating the optimal duration of treatment with amoxicillin for chest indrawing pneumonia; ITIP3: Innovative Treatments in Pneumonia 3, prospective observational study.

Children aged 2-59 months with cough or difficulty breathing were recruited by trained hospital staff during routine intake and screening procedures in the hospitals’ outpatient departments and referred to the study staff for information on the study, written informed consent, and additional screening to determine enrollment eligibility (Figure 2). Screening procedures included assigning a participant an identification number; collecting demographic and contact information and medical history; and assessing eligibility criteria with a targeted physical examination, malaria rapid diagnostic testing, HIV rapid antibody testing, hemoglobin testing, and bronchodilator response testing (if wheezing).

Caregivers of eligible children meeting the case definition (Textbox 1) of fast breathing or chest indrawing pneumonia, who do not meet the eligibility criteria for ITIP1 and ITP2 clinical trials and meet the eligibility criteria for ITIP3 (Textbox 2) were invited to participate by providing written informed consent for enrollment. Final eligibility determination for enrollment depended on the results of the medical history, clinical examination, laboratory testing, appropriate understanding of the study (comprehension assessment checklist), and completion of the enrollment consent process.

Clinical cure was defined as (1) the absence of fast breathing, chest indrawing, severe respiratory distress, hypoxemia, World Health Organization (WHO) Integrated Management of Childhood Illness (IMCI) general danger signs, and fever; (2) cure from pneumonia but failure to complete the initial antibiotic treatment regimen; and (3) cure from pneumonia and completion of the initial antibiotic treatment regimen. In contrast, those who showed a deterioration in their condition or were stable (not improving or deteriorating, prognosis unclear) were not considered to be clinically cured.

**Study definitions.**

Study definitions

- Fast breathing pneumonia: Cough less than 14 days or difficulty breathing AND fast breathing for age
- Chest indrawing pneumonia: Cough less than 14 days or difficulty breathing AND visible indrawing of the chest wall with or without fast breathing for age
- Fast breathing for age: Respiratory rate >50 breaths per minute (for children aged 2 to <12 months of age) or >40 breaths per minute (for children aged >12 months of age)
- Very fast breathing for age: >70 breaths per minute (for children aged 2 to <12 months of age) or >60 breaths per minute (for children aged >12 months of age)
- Severe respiratory distress: Grunting, nasal flaring, and/or head nodding
- Hypoxemia: Arterial oxyhemoglobin saturation < 90% in room air, as assessed noninvasively by a pulse oximeter
- World Health Organization Integrated Management of Childhood Illness general danger signs: Lethargy or unconsciousness, convulsions, vomiting everything, and inability to drink or breastfeed
- Severe acute malnutrition: Weight for height/length < –3 SD, mid-upper arm circumference < 11.5 cm, or peripheral edema
- Severe malaria: Positive malaria rapid diagnostic test with any World Health Organization Integrated Management of Childhood Illness general danger sign, stiff neck, abnormal bleeding, clinical jaundice, or hemoglobinuria
- HIV exposure: Children <24 months of age with an HIV-infected mother
- Serious adverse event: Adverse event that
  - Results in death
  - Is life threatening
  - Requires inpatient hospitalization or prolongation of existing hospitalization
  - Results in persistent or significant disability/incapacity
  - Is a medical event, based on appropriate medical judgment, that may jeopardize the health of the participating child or require medical or surgical intervention to prevent one of the outcomes listed

**Eligibility criteria.**

Inclusion criteria:

- 2-59 months of age
- Cough < 14 days or difficulty breathing
- Excluded from enrollment in ITIP1 and ITIP2 clinical trials due to the presence of any of the following:
  - Severe respiratory distress
  - Hypoxemia
  - Hemoglobin level < 8.0 g/dL, if positive malaria rapid diagnostic test
  - Severe acute malnutrition
  - Severe malaria
  - HIV seropositivity or HIV exposure
- Ability and willingness of child’s caregiver to provide informed consent and to be available for follow-up for the planned duration of the study, including accepting a home visit if he/she fails to return for a scheduled study follow-up visit

Exclusion criteria:

- Stridor when calm
- Possible tuberculosis (coughing for more than 14 days)
- Hemoglobin level < 8.0 g/dL, if negative malaria rapid diagnostic test
- Known allergy to penicillin or amoxicillin
- Receipt of an antibiotic treatment in the 48 hours prior to the study
- Living outside the study area
- Any medical or psychosocial condition or circumstance that, in the opinion of the investigators, would interfere with the conduct of the study or for which study participation might jeopardize the child’s health
- Participation in a clinical study of an investigational product within 12 weeks prior to enrollment or planning to begin participation during this study
- Prior participation in ITIP1, ITIP2, or ITIP3 during a previous pneumonia diagnosis

### Study Procedures

All study procedures are conducted according to the protocol provided in Multimedia Appendix 1 (version 4.0; May 26, 2017). On day 1, after study screening was complete and enrollment informed consent was obtained, the study staff performed the following procedures for enrollment: conducting physical examination, obtaining vaccination history, and collecting additional sociodemographic information. Recruitment, screening, and enrollment occur at the outpatient departments of KCH or BDH, with BDH enrollees transferred to KCH for continued evaluation, observation, and admission, if needed. Hospital observation or admission and follow-up occur solely at KCH.

Each enrolled child receives standard care for their illnesses per Malawian guidelines and KCH protocol and is prospectively followed up by the study staff with scheduled study visits on days 1, 2 (if hospitalized), 6, 14 (in person), and 30 (by phone). All visits occur on the calendar day on which they are initially scheduled or within 24 hours, with the exception of the day 14 visit and the day 30 phone call. The day 14 visit can occur either 2 days before or after day 14, and the day 30 phone call can occur either 2 days before or 14 days after day 30 and still be considered completed within the visit windows. During follow-up visits, the study staff review participants’ medical history since the last study visit and perform a physical examination. In case of a no-show at the scheduled follow-up visits, children are followed up with home visits by the study staff. If a phone call on day 30 is not possible due to no phone in the home, the study staff conducts a home visit to obtain the day 30 outcome information.

### Sample Size

The primary objectives of ITIP3 are to describe the clinical outcomes of children who meet the inclusion criteria for ITIP3 and to investigate whether the percentages of children cured on day 14 among those with either fast breathing or chest indrawing pneumonia and comorbidities such as severe malaria, anemia, severe acute malnutrition, or HIV are lower than those without these comorbidities in the standard of care groups in the concurrent clinical trials. For hypothesis testing, we focused on four high-frequency and high-mortality comorbidities (ie, severe malaria, anemia, severe acute malnutrition, and HIV infection or exposure) within ITIP3. We estimate that the study can enroll 1000 children during the time it is concurrently conducted with the ITIP1 and ITIP2 clinical trials, and we estimate the effect sizes for the primary outcome comparisons for which we have 80% power. We also assume that 1000 children can be enrolled in each of the standard care groups in ITIP1 and ITIP2. With available data from KCH and Malawi, we estimate the prevalence of severe pneumonia to be 10%-24%, severe malaria to be 15%-20%, severe acute malnutrition to be 4%-7%, and HIV seropositivity to be 5%-10%. Based on the estimated treatment failure rates for ITIP1 and ITIP2, we conservatively estimate that clinical cure will be observed in 90%-95% of children in ITIP1 and 85%-90% in ITIP2. We estimate the effect sizes we would be able to see for various comparisons between ITIP3 and ITIP1 or ITIP2, along with different estimated prevalence rates (Table 1). For example, comparing the clinical cure among children with HIV infection or exposure in ITIP3 and those in the standard of care group in ITIP2, if the prevalence of HIV infection or exposure is 10% in ITIP3, we will have 80% power to detect an absolute difference in proportions of 9.9% if at least 90% of the children in ITIP2 are cured and 80.1% of the children with HIV infection or exposure in ITIP3 are cured. Note that the absolute observable difference is largest for severe acute malnutrition, as that outcome is expected to have the lowest prevalence among the outcomes of interest. Nonetheless, we considered all the estimated effect sizes to be clinically relevant.

**Table 1:** Observable effect sizes for comparisons of Innovative Treatments in Pneumonia (ITIP) 3^a^ with ITIP1^b^ or ITIP2^c,d^. N (ITIP1 or ITIP2) = 1000.

Exposure of interest, prevalence of exposure (ITIP3), and N (ITIP3)^e^			Absolute observable difference, %	Prevalence of clinical cure (ITIP1 or ITIP2), %	Prevalence of clinical cure (ITIP3), %
**Severe malaria**						
	**20%**					
		200	5.5	95	89.5
		200	7.2	90	82.8
		200	8.3	85	76.7
	**15%**					
		150	6.3	95	88.7
		150	8.1	90	81.9
		150	9.4	85	75.6
**Anemia**						
	**10%**					
		100	7.6	95	87.4
		100	9.9	90	80.1
		100	11.%	85	73.6
	**7.5%**					
		75	8.8	95	86.2
		75	11.3	90	78.7
		75	13.1	85	72.0
**HIV infection or exposure**						
	**10%**					
		100	7.6	95	87.4
		100	9.9	90	80.1
		100	11.4	85	73.6
	**5%**					
		50	10.9	95	84.1
		50	13.9	90	81.3
		50	15.9	85	69.1
**Severe acute malnutrition**						
	**7%**					
		70	9.1	95	85.9
		70	11.7	90	78.3
		70	13.5	85	71.5
	**4%**					
		40	12.2	95	82.8
		40	15.6	90	74.4
		40	17.8	85	67.2

^a^Prospective observational study.

^b^Randomized controlled clinical trial evaluating the optimal duration of treatment with amoxicillin for fast breathing pneumonia.

^c^Alpha value set to 5% and power set to 80%.

^d^Randomized controlled clinical trial evaluating the optimal duration of treatment with amoxicillin for chest indrawing pneumonia.

^e^Assuming similar values for fast breathing pneumonia and chest indrawing pneumonia to be able to compare the ITIP1 and ITIP2 cohorts.

### Data Collection and Quality Assurance

All study data are collected by clinical study staff using designated source documents or paper-based case report forms (Multimedia Appendix 2), which are then entered into electronic databases. Clinical research data are maintained through a combination of a secure electronic data management system and physical files with restricted access to ensure confidentiality. Data related to the study endpoints will be extracted from the electronic databases for statistical analyses. Two distinct study databases are maintained: the primary study database with study visit data and a database with participating children’s personally identifiable information. The database with identifiable information is maintained separately by the study site, while the designated contract research organization (CRO) maintains the primary study database. To ensure accuracy and completeness, data are routinely reviewed by the site quality control and assurance team as well as the coinvestigators who monitor the site and perform quality assurance reviews, audits, and evaluation of the study safety and progress. Standard Good Clinical Practice (GCP) is followed to ensure accurate, reliable, and consistent data collection.

### Data Management

Primary data management activities, which include data entry and validation, data coding and cleaning, database quality control, disaster recovery plans, preparation and submission of compliance reports, and preparation of final study database, are undertaken by the designated CRO. The on-site study data manager oversees data-related procedures at the study site and is supervised by the CRO data management staff. Data management activities are performed using Clindex Clinical Trial and Data Management software, developed by Fortress Medical Systems (Hopkins, MN). All data management activities are in compliance with the International Council on Harmonization (ICH) GCP E6 (R2), a regulatory sponsoring organization, and institutional requirements for the protection of children and confidentiality of personal and health information.

### Outcomes

The primary endpoint is whether children treated for pneumonia are cured 14 days from diagnosis. Secondary endpoints are treatment regimens in ITIP3; treatment responses of children in ITIP3 in comparison to those in the standard care groups in ITIP1 and ITIP2 as measured by vital signs, oxygen saturation, laboratory test results, length of hospital stay; proportion of children who are rehospitalized or die; and proportion of children in ITIP3 who are clinically cured by day 14 (characteristics such as gender, age, weight, mid-upper arm circumference, HIV status, malaria, and vaccination status were considered).

### Statistical Analysis

Generalized linear models with robust standard error will be used to compare the percentages (absolute risk differences) of children who are clinically cured by day 14 among ITIP3 children who have severe malaria (or are anemic or present with severe acute malnutrition, or are HIV positive or exposed) and children in the standard care groups in ITIP1 or ITIP2. Two-sided tests will be performed, with an alpha value of 0.05. No adjustments will be made for multiple comparisons because of the observational and exploratory nature of this study. If loss to follow-up is higher than 5%, multiple imputations will be considered for sensitivity analyses. The imputations will be performed separately for each cohort using multiple (n=20) hot deck imputations and the child’s age and gender and educational status of the caregiver. Multiple imputation estimates will be combined using the approach by Rubin [8]. Similar linear or generalized linear models will be used for secondary outcome analyses. Where appropriate, we will adjust for gender, age, weight, mid-upper arm circumference, HIV status, malaria, and vaccination status.

### Ethics and Dissemination

#### Ethical Approval and Consent

The study is performed in accordance with the ICH GCP and the Declaration of Helsinki 2008. The study was approved by the Western Institutional Review Board, Washington, USA; the Malawi College of Medicine Research and Ethics Committee, Blantyre, Malawi; and the Malawi Pharmacy, Medicines and Poisons Board. Written informed consent was obtained by trained study staff from at least one of the caregivers of each eligible child prior to enrollment.

#### Possible Risks

There are few potential risks to study participation, given that it is observational in nature and there is no study intervention. Caregivers may feel compelled to enroll in the study in order to receive care for their child within a research setting, which may be perceived to be of a higher quality than the standard care. In order to minimize the risk of coercion, study staff do not recruit participants directly. Instead, hospital clinicians inform caregivers about the study and refer only those who are interested. During the informed consent process, study staff emphasize that the child will receive medical care whether or not he/she is enrolled in the study. Another possible risk involves blood specimen sampling at screening, which can cause pain and bruising at or around the blood draw site. To mitigate this risk, all study staff who collect specimens from children in the study are trained in the appropriate procedures and supervised accordingly. Participation in the study has the potential to compromise care for hospitalized children if study procedures are prioritized above urgent clinical care for acute infections. In order to minimize the possibility that participation in this trial interferes with medical management, KCH staff undertake the clinical management of hospitalized children in accordance with standard procedures. Furthermore, recognizing that some children may not come back for the follow-up visits, our trained study staff locate children who miss their follow-up appointments and conduct these visits in their home.

#### Dissemination

We plan to disseminate the study results in peer-reviewed journals and international conferences, targeting those involved in the clinical care of children in low-resource settings as well as those who develop and advise on policies and guidelines in those settings. This trial is registered with ClinicalTrials.gov (NCT02960919).

## Results

The ITIP project was funded by the Bill and Melinda Gates Foundation (OPP1105080) in April 2014. ITIP3 enrollment started in 2016, and the primary results are expected in 2019.

## Discussion

The following discussion outlines our efforts to safely and efficiently conduct a prospective observational study with the goal of providing informative and generalizable results that are applicable to real-world, nonstudy settings in African low- and middle-income countries.

### Efforts Toward Generalizable Results and Addition to the Literature

The study was specifically developed and pragmatically designed with inclusion and exclusion criteria to allow generalizable results. Children enrolled in this study are diagnosed with pneumonia based on the WHO IMCI clinical guidance. Although microbiological and radiological diagnosis may add improved specificity to the clinical diagnosis of pneumonia, the majority of low-resource settings do not have access to this testing, and children are typically diagnosed based on clinical criteria alone. Children with pneumonia and severe illness or underlying comorbidities are intentionally included to provide additional evidence regarding standard care and outcomes for children with pneumonia and to generate data on the generalizability of the concurrent fast breathing and chest indrawing pneumonia clinical trials. Previous investigations on the management of childhood pneumonia with comorbidities in Malawi examined data prior to scale-up of the *Haemophilus influenzae* type b (Hib) vaccine and relied on clinical diagnosis of comorbidities (eg, malaria and anemia) instead of laboratory test results [9]. Similar studies in other African countries such as Tanzania have been limited by small sample sizes [10], while another larger study in Kenya only focused on mortality risk factors for children with nonsevere pneumonia [11]. Data from ITIP3 may help bridge the gap in data and provide insight regarding the course of childhood pneumonia in this region of Africa.

### Efforts Toward Rigorous Protocol Implementation

Dedicated trained study staff follow-up children enrolled in the study to assure the protocol and standard operating procedures are followed, data are collected without error, and the highest level of safety is provided. Standardized training, supervision, oversight, and testing are undertaken to ensure quality, consistency, and harmonization in study procedures and implementation. Regular site monitoring visits by Save the Children are conducted to assess compliance with human subjects and other research regulations and guidelines, adherence to the study protocol and procedures, quality and accuracy of data collected, and quality of care and child safety.

### Limitations and Bias

A limitation to this study and a potential source of bias is loss to follow-up. To minimize loss to follow-up, caregivers are provided clear follow-up instructions as well as called the afternoon before their visits to remind them to visit the following day. A travel stipend for all follow-up visits is provided. In addition, children are followed up with home visits upon a missed visit. An additional limitation related to follow-up is that children in this observational study are followed up less frequently and have different assessment time points than those who are enrolled in the two clinical trials for fast breathing pneumonia and chest indrawing pneumonia. The three study protocols also have differences in the eligibility criteria and treatment regimens. These differences, in addition to the different follow-up schedules, will present some challenges for analyses and interpretation of results. Where possible, we will attempt to adjust for baseline characteristics (not in the causal pathway) as well as perform sensitivity analyses. Nevertheless, the potential for unmeasured confounders cannot necessarily be overcome or assessed. Other limitations to this study are the clinical diagnosis of pneumonia, rather than a microbiological or radiological diagnosis, and the limited follow-up duration of 30 days. All study staff receive rigorous training in the WHO IMCI classification of pneumonia; however, no microbiological or radiological tests are routinely undertaken unless clinically indicated. Of note, this was a pragmatic design decision, as in standard clinical care in this setting, microbiological or radiological tests are not typically undertaken unless clinically indicated. In addition, it is possible that we are missing longer-term consequences after treatment, given the limited follow-up period of 30 days.

## Appendix

Multimedia Appendix 1 ITIP3 protocol.

Multimedia Appendix 2 ITIP1 and ITIP2 follow-up visit schedule.

## Abbreviations

BDH: Bwaila District Hospital
CRO: contract research organization
GCP: good clinical practice
Hib: Haemophilus influenzae type b
ICH: International Council on Harmonisation
ITIP: Innovative Treatments in Pneumonia
IMCI: integrated management of childhood illness
KCH: Kamuzu Central Hospital
MUAC: mid-upper arm circumference
OR: odds ratio
WHO: World Health Organization
