# Title Correction: Clinical Outcomes of Pneumonia and Other Comorbidities in Children Aged 2-59 Months in Lilongwe, Malawi: Protocol for the Prospective Observational Study “Innovative Treatments in Pneumonia”

**DOI:** 10.2196/16007

**Published:** 2019-08-26

**Authors:** Amy Sarah Ginsburg, Susanne May, Evangelyn Nkwopara, Gwen Ambler, Eric D McCollum, Tisungane Mvalo, Ajib Phiri, Norman Lufesi

**Affiliations:** 1 Save the Children Fairfield, CT United States; 2 Department of Biostatistics University of Washington Seattle, WA United States; 3 PATH Seattile, WA United States; 4 Eudowood Division of Pediatric Respiratory Sciences John Hopkins School of Medicine Baltimore, MD United States; 5 University of North Carolina Project: Lilongwe, Central Region Lilongwe Malawi; 6 Department of Paediatrics and Child Health College of Medicine University of Malawi Blantyre Malawi; 7 Ministry of Health, Republic of Malawi Lilongwe Malawi

The title of this paper (JMIR Res Protoc 2019;8(7):e13377) has been changed from:

“Clinical Outcomes of Pneumonia and Other Comorbidities in Children Aged 2-59 Months in Lilongwe, Malawi: Protocol for the Prospective Observational Study “Innovative Treatments for Pneumonia”

to:

Clinical Outcomes of Pneumonia and Other Comorbidities in Children Aged 2-59 Months in Lilongwe, Malawi: Protocol for the Prospective Observational Study “Innovative Treatments in Pneumonia”

The correction will appear in the online version of the paper on the JMIR website on August 26, 2019, together with the publication of this correction notice. Because this was made after submission to PubMed, PubMed Central, and other full-text repositories, the corrected article also has been resubmitted to those repositories.

