# Supplementary material for: Clinical Outcomes of Pneumonia and Other Comorbidities in Children Aged 2-59 Months in Lilongwe, Malawi: Protocol for the Prospective Observational Study “Innovative Treatments in Pneumonia”
Source: JMIR Res Protoc. 2019 Jul 29;8(7):e13377. doi: 10.2196/13377 (PMC6690162; doi:10.2196/13377)
Supplement: Multimedia Appendix 1 [file resprot_v8i7e13377_app1.pdf]

Title: Innovative Treatments in Pneumonia (ITIP) 3

Subtitle: Prospective, observational study of clinical outcomes among children 2 to 59 months of age with childhood pneumonia and other co-morbidities in Lilongwe, Malawi who have been excluded from pneumonia clinical trials

Sponsored by:

Save the Children Federation, Inc.

Version 4.0
May 26, 2017

Confidentiality Statement

This document is confidential and is to be distributed for review only to investigators, potential investigators, consultants, study staff, applicable regulatory authorities, and applicable independent ethics committees or institutional review boards. The contents of this document shall not be disclosed to others without written authorization from Save the Children (or others, as applicable), unless it is necessary to obtain informed consent from potential study participants.

Innovative Treatments in Pneumonia 3

TABLE OF CONTENTS

TABLE OF CONTENTS 2
ABBREVIATIONS AND ACRONYMS 4
INVESTIGATORS 5
EXECUTIVE SUMMARY 7
1 BACKGROUND AND INTRODUCTION 11
2 RATIONALE 11
3 STUDY HYPOTHESIS, OBJECTIVES AND ENDPOINTS 12
- Study Hypothesis 12
- Study Objectives 12
- Study Endpoints 14
4 METHODOLOGY 15
4.1 STUDY DESIGN 15
4.2 STUDY SITE 15
4.3 STUDY POPULATION 16
- Study Population Overview 16
- Participant Eligibility 16
- Inclusion Criteria 17
- Exclusion Criteria 17
4.4 STUDY PERIOD 18
4.5 SAMPLE SIZE 18
4.6 STUDY PROCEDURES 20
- Recruitment 20
- Screening 20
- Informed Consent 21
- Enrollment Visit 22
- Management of Study Participants During Hospitalization 22
- Follow-Up Visits 23
- Missed Visits 23
- Interim Contacts and Visits 24
- Withdrawal and Early Termination 24
- Study Termination Visit 24
- Biohazard Containment 24
4.7 STUDY MEDICATION USE 24
4.8 DATA COLLECTION 24
- Case Report Forms 25
- Source Documents 25
4.9 DATA MANAGEMENT 25
- Data Access 26

- Data Storage 26
- External Study Monitoring 26

4.10 ASSESSMENTS AND REPORTING 26
- Monitoring 26
- Adverse Events 27
- Serious Adverse Event 27
- Study Discontinuation 27

5 STATISTICAL DESIGN AND ANALYSIS 28

5.1 DATA ANALYSIS 28
- Overview and General Design 28
- Objectives and Endpoints 28
- Analytical Methodology 29

5.2 RESULT PRESENTATION 29

5.3 DISSEMINATION OF RESULTS 29

6 ETHICAL CONSIDERATIONS AND CONSENT 29
- Principles for Clinical Research 30
- Institutional Review Boards (IRBs) and Independent Ethics Committees (IECs) 30
- Informed Consent 30
- Risks to Participants 30
- Protection against Risks 31
- Benefits to Participants 31
- Participant Confidentiality 32
- Participant Reimbursement 32
- Storage of Specimens 32

7 POSSIBLE CONSTRAINTS 32

8 REQUIREMENTS AND TRAINING 33

9 BUDGET 33
- Budgetary Estimates 33
- Budget Justification 34

10 REFERENCES 34

APPENDICES 38

APPENDIX I: SCHEDULE OF STUDY VISITS AND EVALUATIONS 38

APPENDIX II: SAMPLE COLLECTION AND LABORATORY EVALUATIONS 38

APPENDIX III: SAMPLE SIZE CALCULATIONS 39

APPENDIX IV: STUDY REQUIREMENTS AND TRAINING 44

APPENDIX V: STUDY SENSITIZATION/RECRUITMENT SCRIPT 46

Innovative Treatments in Pneumonia 3

ABBREVIATIONS AND ACRONYMS

AE	adverse event
BDH	Bwaila District Hospital
BMGF	Bill & Melinda Gates Foundation
COM	College of Medicine
COMREC	College of Medicine Research and Ethics Committee
CRF	case report form
CRO	contract research organization
DT	dispersible tablets
GCP	Good Clinical Practices
HIV	Human Immunodeficiency Virus
ICF	informed consent form
ICH	International Conference on Harmonisation of Technical Requirements for Registration of Pharmaceuticals for Human Use
ID	identification
IEC	independent ethics committee
IMCI	Integrated Management of Childhood Illness
IRB	institutional review board
ITIP	Innovative Treatments in Pneumonia
KCH	Kamuzu Central Hospital
LAR	legally authorized representative
LPI	local principal investigator
MOH	Ministry of Health
mRDT	malaria rapid diagnostic test
OPD	outpatient department
PI	principal investigator
SAE	serious adverse event
SAM	severe acute malnutrition
SCUS	Save the Children Federation, Inc., United States
SCI	Save the Children International, Malawi
SOP	standard operating procedure(s)
UNC	University of North Carolina Project Lilongwe Trust
UW	University of Washington
WHO	World Health Organization

Innovative Treatments in Pneumonia 1

INVESTIGATORS

Principal investigator (PI):
Amy Ginsburg, MD, MPH
Senior Advisor, Child Health
Save the Children Federation, Inc.

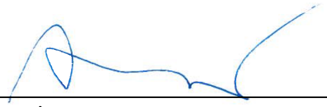

PI signature   26-May-2017
Date

Local co-PIs:
Ajib Phiri, MD
Senior Academic Staff, Department of Pediatrics and Child Health
Malawi College of Medicine

Tisungane Mvalo, MB BS, FC Paed
Consultant Pediatrician and Clinical Research Investigator
University of North Carolina (UNC) Project Lilongwe Trust, Malawi

Co-investigators:
Susanne May, PhD
Associate Professor, Department of Biostatistics
University of Washington

Eric McCollum, MD
Assistant Professor, Department of Pediatrics
Johns Hopkins School of Medicine

Norman Lufesi, CO, M.Phil.
Program Manager, Acute Respiratory Infection & Emergency Triage Assessment and Treatment
Malawi Ministry of Health

PARTICIPATING INSTITUTIONS

**Sponsor:**	Save the Children Federation, Inc.<br>501 Kings Highway East, Suite 400<br>Fairfield, CT 06825	
**Monitor:**	Triclinium Clinical Trial Project Management (Pty) Ltd.<br>135 West Street, Suite 400<br>Sandown 2196<br>South Africa	
**Medical Officers:**	Amy Ginsburg, MD, MPH<br>Save the Children Federation, Inc.<br>501 Kings Highway East, Suite 400<br>Fairfield, CT 06825<br>+1-203-221-4000	Janetta Strauss, MBChB, MPharmMed<br>Triclinium<br>135 West Street, Suite 400<br>Sandown 2196 South Africa<br>+27-11-883-0205
**Clinical Study Site:**	Kamuzu Central Hospital<br>Box 149<br>Lilongwe 265 (Area 33)<br>Malawi<br>+265-1-75-3555	
**Study Operations:**	University of North Carolina Project Lilongwe Trust	
**Data Center:**	Triclinium Clinical Trial Project Management (Pty) Ltd.	
**Statistical Support:**	University of Washington	
**Local Collaborators:**	College of Medicine at the University of Malawi<br>Malawi Ministry of Health<br>Kamuzu Central Hospital<br>Bwaila District Hospital<br>Save the Children International – Malawi Country Office	
**Funding Agency:**	Bill and Melinda Gates Foundation	

EXECUTIVE SUMMARY

Problem to be studied: Pneumonia is responsible for more than one in five child deaths around the globe. Each year, approximately 935,000 children die before their fifth birthdays due to pneumonia, more than the number of under-five deaths that result from human immunodeficiency virus (HIV), tuberculosis, and malaria combined. In addition to preventing pneumonia, there is a critical need to provide greater access to appropriate and effective treatment.

A prospective observational study will be conducted to assess the clinical outcomes of children 2 to 59 months of age with both pneumonia and other co-morbidities in an effort to generate data on pneumonia treatment outcomes among higher risk African children post introduction of *Haemophilus influenzae* and pneumococcal conjugate vaccines. This observational study will be performed in conjunction with the Innovative Treatments in Pneumonia (ITIP) clinical trials, ITIP1 and ITIP2. The primary aim of the ITIP1 and ITIP2 clinical trials is to provide scientific evidence assessing the optimal duration of treatment with amoxicillin dispersible tablets (DT) for fast-breathing and chest-indrawing childhood pneumonia (but without major other co-morbidities) in a malaria endemic setting in Africa. Thus, these two clinical trials will be conducted among immunocompetent children 2 to 59 months of age residing in a malaria endemic region of Malawi: 1. one examining the relative effectiveness of placebo compared to 3-day amoxicillin DT treatment for fast-breathing pneumonia (ITIP1); and 2. the other comparing 3-day to 5-day amoxicillin DT treatment for chest- indrawing pneumonia (ITIP2). The prospective observational study, ITIP3, will enroll children with pneumonia who are excluded from the two clinical trials because of other co-morbidities.

Given the paucity of data from Africa, African-based research is necessary to establish optimal management of childhood pneumonia in the region. With the expressed support of the Malawi Ministry of Health (MOH) and in collaboration with external experts from the University of Washington (UW) and the Johns Hopkins School of Medicine, Save the Children Federation, Inc., United States (SCUS) will work closely with investigators from the College of Medicine (COM) at the University of Malawi and University of North Carolina (UNC) Project Lilongwe Trust to provide additional valuable evidence on the standard care and outcomes for children 2 to 59 months of age with pneumonia presenting to a tertiary hospital setting in Malawi who are most at risk for mortality or have other complications.

Type of research: The proposed approach involves conducting a prospective observational study assessing the clinical outcomes of children 2 to 59 months of age with both pneumonia and other co-morbidities presenting to a tertiary hospital outpatient setting in Malawi, Africa who are most at risk for mortality or have other complications and thus, are typically excluded from childhood pneumonia studies.

Objective: The primary objectives of this study are to determine the clinical outcomes of children with pneumonia and other co-morbidities aged 2 to 59 months who are excluded from the ITIP1 and ITIP2 clinical trials of childhood pneumonia treatment and meet inclusion criteria for ITIP3, and to investigate whether the percentages of children cured at day 14 among those with either fast-breathing pneumonia or chest-indrawing pneumonia AND co-morbidities such as

severe malaria, anemia, severe acute malnutrition (SAM), or HIV are lower than those without these co-morbidities in the standard of care arms in the concurrent clinical trials.

Methodology: The study will enroll 1000 children 2 to 59 months of age with pneumonia and other co-morbidities presenting to Kamuzu Central Hospital (KCH) or Bwaila District Hospital (BDH) in Lilongwe, Malawi who are excluded from ITIP1 and ITIP2 and meet inclusion criteria for ITIP3. Each child will receive standard of care per Malawian guidelines and/or KCH ward protocol (e.g., 5 days of oral amoxicillin or, for severe pneumonia, parenteral ampicillin/ penicillin and gentamicin) and will be followed for 14 days with regular study visits at days 2 (if hospitalized as per KCH standard of care), 6, 14 and 30 (by phone) after enrollment.

Expected findings and their dissemination: We will describe clinical outcomes for ITIP3. We will compare clinical outcomes between ITIP3 children and children in the standard of care arms of ITIP1 and ITIP2 and hypothesize (under the alternative): a) the percentage of children who are clinically cured by day 14 will be lower among children with fast-breathing pneumonia and severe malaria in ITIP3 than among children in the standard care arm of ITIP1; b) the percentage of children who are clinically cured by day 14 will be lower among children with fast-breathing pneumonia and SAM in ITIP3 than among children in the standard care arm of ITIP1; c) the percentage of children who are clinically cured by day 14 will be lower among children with fast-breathing pneumonia and HIV in ITIP3 than among children in the standard care arm of ITIP1; d) the percentage of children who are clinically cured by day 14 will be lower among children with fast-breathing pneumonia and anemia in ITIP3 than among children in the standard care arm of ITIP1; e) the percentage of children who are clinically cured by day 14 will be lower among children with chest-indrawing pneumonia and severe malaria in ITIP3 than among children in the standard care arm of ITIP2; f) the percentage of children who are clinically cured by day 14 will be lower among children with chest-indrawing pneumonia and SAM in ITIP3 than among children in the standard care arm of ITIP2; and g) the percentage of children who are clinically cured by day 14 will be lower among children with chest-indrawing pneumonia and HIV in ITIP3 than among children in the standard care arm of ITIP2; h) the percentage of children who are clinically cured by day 14 will be lower among children with chest-indrawing pneumonia and anemia in ITIP3 than among children in the standard care arm of ITIP2. We will also conduct exploratory investigations on whether there is a differential treatment response by gender, age weight, MUAC, HIV status, malaria, and vaccination status among children enrolled in ITIP3. We will also determine post-discharge treatment outcomes amongst hospitalized children included in ITIP3. Findings from this study will be disseminated through a peer-reviewed journal and shared with the scientific community and local stakeholders.

PROTOCOL OUTLINE

Title: **Innovative Treatments in Pneumonia (ITIP) 3**: Prospective, observational study of clinical outcomes among children 2 to 59 months of age with childhood pneumonia and other co-morbidities in Lilongwe, Malawi who have been excluded from pneumonia clinical trials

Sponsor: Save the Children Federation, Inc.

Collaborating Organizations:
Malawi Ministry of Health
College of Medicine at the University of Malawi
Kamuzu Central Hospital
Bwaila District Hospital
University of North Carolina Project Lilongwe Trust
University of Washington
Save the Children International, Malawi Country Office

Funding Source: Bill and Melinda Gates Foundation

Study Medication: Standard of care per Malawian guidelines and/or Kamuzu Central Hospital (KCH) ward protocol (e.g., 5 days of oral amoxicillin or, for severe pneumonia, parenteral ampicillin/ penicillin and gentamicin)

Rationale: Build evidence regarding the clinical outcomes of children with pneumonia and other co-morbidities whom are excluded from clinical trials of potential new treatment regimens in a malaria-endemic setting in Africa

Population: 1000 children ages 2 to 59 months of age with pneumonia and other co-morbidities who are excluded from ITIP1 and ITIP2 and meet inclusion criteria for ITIP3

Schema:

	N	Day 1	Day 2	Day 6	Day 14	Day 30
Cohort	1000	X	X*	X	X	X

*If hospitalized per KCH ward protocol

Objectives:

1. Primary: clinical outcomes for ITIP3 and comparison of clinical outcomes in ITIP3 with children in standard of care groups in ITIP1 and ITIP2
2. Secondary: treatment regimens, cofactors of response

Endpoints:

1. Primary: clinical outcomes at day 14
2. Secondary: treatment regimens, treatment responses (such as vital status and hospital length of stay), proportion of clinical outcomes by gender, age, weight, MUAC, HIV status, malaria, and vaccination status.

**Timeline:**	Projected duration of enrollment is about 24 months (simultaneous recruitment with ITIP1 and ITIP2).<br>All children will be followed for 30 days after enrollment.

Innovative Treatments in Pneumonia 3

1 BACKGROUND AND INTRODUCTION

The burden of childhood pneumonia remains high, and improved access to effective treatment saves lives. Because vaccines cannot prevent all episodes of pneumonia and because pneumonia incidence remains unacceptably high, there is an urgent need to focus on effective and affordable treatment. In low-resource settings, the World Health Organization's (WHO) integrated management of childhood illness (IMCI) guidelines diagnose pneumonia by identifying fast-breathing and chest-indrawing.[1] Children with fast- breathing and/or chest-indrawing are considered to have "pneumonia" with outpatient treatment recommended; children with additional symptoms are classified with "severe pneumonia" and the guidelines specify inpatient treatment.[8]

Based on recently revised WHO guidelines, amoxicillin is recommended as the first-line treatment for both subcategories of the "pneumonia" classification.[9][10] In settings of low HIV prevalence, WHO recommendations are for 3 days of twice daily dosing for the children in the fast-breathing subcategory and for 5 days of twice daily dosing for children in the chest-indrawing subcategory. Children who are HIV positive or exposed are recommended to receive 5 days of twice daily dosing.

Pneumonia with any WHO general danger sign is now considered "severe pneumonia" and is treated with injectable therapy: for children aged 2 to 59 months, parenteral ampicillin (or penicillin) and gentamicin as first-line treatment. Ceftriaxone is recommended as a second-line treatment for those who have failed first-line treatment. Ampicillin (or penicillin when ampicillin is not available) plus gentamicin or ceftriaxone are recommended as a first-line antibiotic regimen for HIV-infected and -exposed infants and for children under 5 years of age with chest-indrawing pneumonia or severe pneumonia. For HIV-infected and -exposed infants and for children with chest-indrawing pneumonia or severe pneumonia, who do not respond to treatment with ampicillin or penicillin plus gentamicin, ceftriaxone alone is recommended for use as second-line treatment. Empiric cotrimoxazole treatment for suspected *Pneumocystis jirovecii* pneumonia (PCP) is recommended as an additional treatment for HIV-infected and -exposed infants aged from 2 months up to 1 year with chest-indrawing or severe pneumonia.

We plan to conduct a prospective, observational study of children excluded from ITIP1 and ITIP2 because they were diagnosed with severe pneumonia or had other co-morbidities or complicating factors such as severe malaria, anemia, SAM or HIV infection or exposure, and thus, can provide data on pneumonia treatment outcomes among higher risk African children post introduction of *Haemophilus influenzae* and pneumococcal conjugate vaccines.

2 RATIONALE

There is a critical need for African-specific data, as countries in Africa, including Malawi, endeavor to put into place evidence-based policies and treatment guidelines informed by the local context. To learn more about the treatment of and recovery from childhood pneumonia, especially among those with co-morbidities or severe presentations, and to understand how children with such co-morbidities who are typically excluded from clinical trials compare to the children enrolled in clinical trials evaluating treatment regimens, this prospective study proposes to enroll a cohort of children who are excluded from ITIP1 and ITIP2 and follow each child for up to 30 days. The observational study described in this protocol is intended to provide information that could augment the knowledge gained by the trials results, and therefore, the treatment of childhood pneumonia in low resource settings.

We anticipate the findings from the ITIP3 observational study will help us better understand the clinical course and follow-up of children with pneumonia and co-morbidities, or those with more severe presentations of pneumonia. The health benefits will be close monitoring and observation of children with pneumonia and co-morbidities, or those with more severe presentations of pneumonia, rather than providing that oversight only to ITIP1 and ITIP2 study participants. Of note, the disease presentations of children enrolled in the ITIP3 observational study are expected to be very different from those of children enrolled in the ITIP1 and ITIP2 clinical trials. ITIP1 and ITIP2 are targeting very specific subsets of pneumonia classification. We anticipate that the disease presentations in ITIP3 will be more severe than those in the ITIP1 and ITIP2 clinical trials. As such, the results from the ITIP1 and ITIP2 clinical trials will not have a direct impact on children enrolled in the ITIP3 observational study. Because of the substantial differences in disease presentations and illness severities, we do not anticipate any reason why upon completion of the ITIP1 and ITIP2 clinical trials, regardless of the results, that we would conclude that children enrolled in ITIP3 should have been enrolled in the ITIP1 and ITIP2 clinical trials. Without ITIP3, the findings from the ITIP1 and ITIP2 trials could not be applicable to all Malawian children with pneumonia. We can envision a potential future trial, depending on the results of the ITIP3 observational study, focusing on the children represented by the ITIP3 population. We would expect the treatment approaches and options to vary substantially from ITIP1 and ITIP2 treatment approaches and options due to the co-morbidities and more severe presentations in this population.

3 STUDY HYPOTHESIS, OBJECTIVES AND ENDPOINTS

- **Study Hypothesis** (stated under the alternative)

  The primary objectives are to describe clinical outcomes for children with pneumonia in ITIP3, and to test the hypotheses that the proportion of children clinically cured will be lower in ITIP3 than for children in the standard of care arms of ITIP1 and ITIP2 who might have some, but not all of the same co-morbidities. For the hypothesis testing, we focus on four high frequency and high mortality co-morbidities: severe malaria, anemia, SAM, and HIV infection or exposure.

- **Study Objectives**

The broad objective of this study is to assess the clinical outcomes in a tertiary hospital setting in Malawi, Africa for children 2 to 59 months of age with pneumonia who are most at risk for mortality or have other complications and thus, who may be excluded from childhood pneumonia studies.

- Primary Objectives
  - To determine the clinical outcomes of children with pneumonia and other co-morbidities aged 2 to 59 months who are excluded from the clinical trials of childhood pneumonia treatment ITIP1 and ITIP2 and meet inclusion criteria for ITIP3.
  - To compare the clinical outcomes of children with fastbreathing pneumonia and severe malaria or anemia or SAM or HIV in ITIP3 with those of children in the standard care arm of ITIP1. *Hypotheses below are all stated under the alternative*.
    - *Hypothesis:* The percentage of children who are clinically cured by day 14 will be lower among children with fast-breathing pneumonia and severe malaria in ITIP3 than among children in the standard care arm of ITIP1.
    - *Hypothesis:* The percentage of children who are clinically cured by day 14 will be lower among children with fast-breathing pneumonia and anemia in ITIP3 than among children in the standard care arm of ITIP1.
    - *Hypothesis:* The percentage of children who are clinically cured by day 14 will be lower among children with fast-breathing pneumonia and SAM in ITIP3 than among children in the standard care arm of ITIP1.
    - *Hypothesis:* The percentage of children who are clinically cured by day 14 will be lower among children with fast-breathing pneumonia and HIV infection or exposure in ITIP3 than among children in the standard care arm of ITIP1.
  - To compare the clinical outcomes of children with chest-indrawing pneumonia and severe malaria, or anemia, or SAM or HIV in ITIP3 with those of children in the standard care arm of ITIP2.
    - *Hypothesis:* The percentage of children who are clinically cured by day 14 will be lower among children with chest-indrawing pneumonia and severe malaria in ITIP3 than among children in the standard care arm of ITIP2.
    - *Hypothesis:* The percentage of children who are clinically cured by day 14 will be lower among children with chest-indrawing pneumonia and anemia in ITIP3 than among children in the standard care arm of ITIP2.
    - *Hypothesis:* The percentage of children who are clinically cured by day 14 will be lower among children with chest-indrawing pneumonia and SAM in ITIP3 than among children in the standard care arm of ITIP2.
    - *Hypothesis:* The percentage of children who are clinically cured by day 14 will be lower among children with chest-indrawing pneumonia and HIV infection or exposure in ITIP3 than among children in the standard care arm of ITIP2.

Assessment of groups with high frequency and high mortality co-morbidities:

- Malaria rapid diagnostic test (mRDT) result and signs of severe malaria stratified by anemia status.

- SAM (i.e., weight for height/length < -3 standard deviation [SD], mid-upper arm circumference [MUAC] <115 millimeter [mm], or edema).
- HIV-positive rapid test result or HIV-exposure due to maternal HIV-positive rapid test result.
- Anemia defined as hemoglobin <8.0 g/dL.

- Secondary Objectives
  - To categorize and describe the treatment regimens prescribed for children with pneumonia and other co-morbidities in ITIP3.
  - To describe treatment responses over a period of 14 days for children with pneumonia, including vital signs, oxygen saturation, laboratory results, length of hospital stay, re-hospitalization and deaths for ITIP3.
  - To compare treatment responses over a period of 14 days for children with pneumonia between ITIP3 and children in the standard of care arms in ITIP1 and ITIP2, including vital signs, oxygen saturation, laboratory results, length of hospital stay, re-hospitalization and deaths.
  - To conduct exploratory investigations on whether there is a differential treatment response by gender, age, weight, MUAC, HIV-status, malaria, and vaccination status among children enrolled in ITIP3.

- **Study Endpoints**

  - Primary Endpoints

    Clinical outcome after 14 days from diagnosis of children treated for pneumonia, defined as:
    - Clinically cured – absence of fast-breathing, chest-indrawing, hypoxemia, severe respiratory distress (e.g., presence of grunting, nasal flaring, head nodding, or severe chest-indrawing), WHO danger signs (i.e. lethargy or unconsciousness, convulsions, vomiting everything, or inability to drink or breastfeed), and fever:
      - Cured but failed initial antibiotic treatment regimen
      - Cured and did not fail initial antibiotic treatment regimen
    - Not cured:
      - Deteriorating
      - Stable (not improving or deteriorating, prognosis unclear)

- Secondary Endpoints
    - Treatment regimens prescribed for pneumonia for children enrolled in ITIP3, including dosage, route, and duration as well as any change in initial treatment regimen (e.g., oxygen, continuous positive airway pressure (CPAP) antibiotics, fluids).
    - Treatment responses over a period of 14 days for children in ITIP3, with comparisons to children in the standard of care arms in ITIP1 and ITIP2, as measured by:
        - Vital signs (e.g., respiratory rate, heart rate, temperature)
        - Oxygen saturation levels on room air
        - Available laboratory values (e.g., hemoglobin) and radiography findings (e.g., chest radiograph)
        - Length of hospital stay
        - Proportion of children who are re-hospitalized or die
    - Proportion of various clinical outcomes in ITIP3 by gender, age (e.g., less than 1 year or between 12 and 59 months), weight, MUAC, HIV-status, malaria, and vaccination status (e.g., *Haemophilus influenzae* type b [Hib] and pneumococcal conjugate vaccine [PCV] doses received or fully vaccinated).

4 METHODOLOGY

4.1 STUDY DESIGN

This project involves a prospective, observational study of clinical outcomes among 1000 children 2 to 59 months of age with childhood pneumonia and other co-morbidities presenting to KCH or Bwaila District Hospital (BDH) in Lilongwe, Malawi who have been excluded from pneumonia clinical trials ITIP1 and ITIP2 and meet inclusion criteria for ITIP3. Each child will receive standard of care per Malawian guidelines and/or KCH ward protocol (e.g., 5 days of oral amoxicillin or, for severe pneumonia, parenteral ampicillin/ penicillin and gentamicin) and will be followed for 14 days with regular study visits at days 2 (if hospitalized as per KCH standard of care), 6, 14 and 30 (by phone) after enrollment.

4.2 STUDY SITE

The SCUS PI; the local co-PIs (LPIs) from the Malawi COM and UNC; and the team of co-investigators will conduct the research at KCH and BDH in Lilongwe. A 750-bed government facility, KCH is the primary referral hospital for the central region of Malawi, serving a population of approximately 5 million. Up to 30 or 40 children 5 years of age or younger with fast-breathing pneumonia are seen each day in the outpatient department (OPD) at KCH during the peak pneumonia season. The KCH pediatric department alone admits around 22,000 children per year. Over 100 children 5 years or age or younger with chest-indrawing pneumonia are admitted on average each month at KCH, with as many as 260 admissions a month during peak pneumonia season.

One of the major medical training institutions in Malawi, KCH has four full-time on-call pediatricians, four part-time pediatricians, three medical officers, three to six medical interns, 12

full-time clinical officers, and 45 nurses on staff. In the OPD, two to three clinicians with the support of two to three health surveillance assistants manage the triage area. In addition to the triage area, there are also emergency/resuscitation, priority, and low-risk areas in the OPD. The hospital has a functioning laboratory unit and a radiology unit that is capable of conducting chest radiographs (including a mobile unit, which is housed in the pediatric ward), ultrasounds, and computed tomography scans.

BDH is the district hospital for Lilongwe and is located in the central business district of Lilongwe. Approximately 200 to 250 children present to the BDH OPD per day. There are no inpatient facilities for children at BDH, and those requiring inpatient care are referred to KCH.

4.3 STUDY POPULATION

- **Study Population Overview**

  Although KCH draws from a large catchment area in the central region of Malawi, children eligible for this study are to be from the Lilongwe District. Malawi is ranked 174th in the United Nations Development Programme's human development index, and almost 89% of the working population earns less than $2USD a day.[44] Lilongwe includes large peri-urban settlements with crowded living conditions and without adequate sanitation infrastructure. Overall, the nation's adult literacy rate is 61%. Life expectancy at birth is 55.31 years and the under-five mortality rate is 71 deaths per 1000 children.

  Malaria is endemic in Malawi with the highest prevalence of malaria parasitaemia in children between 6 and 36 months of age (60.1%).[45] The incidence of malaria in the area is highest during the rainy season, between December and April each year. Malawi's adult HIV prevalence was estimated to be 10.3% in 2013. There were 170,000 estimated HIV-positive children 14 and younger. HIV is more prevalent in urban communities than rural areas.[46]

  We expect study participants to be representative of the ethnic demographics in the area. We anticipate enrolling equal numbers of female and male children for a total participant population of 1000 volunteers for this study.

- **Participant Eligibility**

  Study participants will be children 2 to 59 months of age who present to KCH or BDH with fast-breathing pneumonia or chest-indrawing pneumonia and other co-morbidities, or severe pneumonia AND are excluded from ITIP1 and ITIP2. Volunteer families will be recruited and screened, those whose children are determined to be eligible, based on the inclusion/exclusion criteria, will be enrolled in the study and followed for 30 days. Recruitment, screening and enrollment can occur at KCH or BDH. Hospital observation or admission, and follow-up will occur at KCH. Final eligibility determination will depend on the results of the medical history, clinical examination, appropriate understanding of the study and completion of the consent process.

Case definition of pneumonia:

- Cough <14 days and/or difficulty breathing AND one of the following:
    1. Respiratory rate $\geq$50 breaths/minute (for children 2 to <12 months of age) or $\geq$40 breaths/minute (for children $\geq$12 months of age); and/or visible chest-indrawing, with or without fast-breathing plus a general clinical danger sign.
    2. Respiratory rate $\geq$50 breaths/minute (for children 2 to <12 months of age) or $\geq$40 breaths/minute (for children $\geq$12 months of age); and/or visible chest-indrawing, with or without fast-breathing plus another exclusion criteria from ITIP1 or ITIP2.
    3. Hypoxia (SaO_2 < 90% on room air, as assessed by a pulse oximeter).
    4. Severe respiratory distress (e.g., presence of grunting, nasal flaring, head nodding, or severe chest-indrawing).

1. Inclusion Criteria

- Male or female, 2 to 59 months of age.
- Excluded from enrollment in ITIP1 and ITIP2 clinical trials due to presence of any of the following:
    - Hypoxia (SaO_2 < 90% on room air, as assessed by a pulse oximeter).
    - Severe respiratory distress (e.g., presence of grunting, nasal flaring, head nodding, or severe chest-indrawing).
    - Severe malaria, classified by WHO guidelines on hospital care for children, including a positive malaria rapid antibody test result.
    - Severe anemia, classified by WHO IMCI guidelines (i.e., severe palmar pallor) only if a positive malaria rapid antibody test result.
    - SAM (i.e., weight for height/length < -3 SD, MUAC <115, or edema).
    - HIV-1 seropositivity or HIV-1 exposure, assessed as follows:
        - An HIV-positive result upon rapid antibody testing.
        - If a child is less than 24 months of age and has an HIV-negative result upon rapid antibody test documented from the past three months, the child's biological mother's HIV status will need to be assessed. If the mother is HIV-positive, the child will be included. If the mother does not have documentation of an HIV-negative test result from the past 3 months, she will be tested via rapid antibody testing to determine the child's eligibility for this study.
- Ability and willingness of child's caregiver to provide informed consent and to be available for follow-up (in the inpatient ward, returning to KCH for a scheduled study follow-up visit, and by phone) for the planned duration of the study.

2. Exclusion Criteria

- Possible tuberculosis (coughing for more than 14 days).
- Stridor when calm.
- Severe anemia, classified by WHO IMCI guidelines (i.e., severe palmar pallor) if a negative malaria rapid antibody test result.

- Known allergy to penicillin or amoxicillin.
- Receipt of an antibiotic treatment in the 48 hours prior to the study based on caregiver's self-report and/or documentation in child's medical record.
- Living outside Lilongwe urban area, the study catchment area.
- Any medical or psychosocial condition or circumstance that, in the opinion of the investigators, would interfere with the conduct of the study or for which study participation might jeopardize the child's health.
- Participation in a clinical study of an investigational product within 12 weeks prior to enrollment or planning to begin participation during this study.
- Prior participation in ITIP1, ITIP2 or ITIP3 during a previous pneumonia diagnosis.

4.4 STUDY PERIOD

Each child will be followed for 30 days after enrollment. Projected duration of enrollment is anticipated to be about 24 months. The high volume of children presenting to the OPD each day with pneumonia is expected to exceed the capacity of study staff to adequately assess each child for this study, so a maximum of 10 children will be enrolled each day. To avoid potential selection bias, each day children will be screened for enrollment in a sequential manner, as much as possible. Children that are not assessed for this study will receive the standard of care at KCH or BDH, which includes antibiotics, a case-by-case assessment for hospitalization, and treatment of any co-infections.

The funding for this study is for three years, through December 31, 2018. This period includes the time required to prepare the necessary documents for the study, train all study personnel, initiate the study site, conduct the study and all data collection procedures, clean and analyze the data, and prepare the results for publication and presentation.

4.5 SAMPLE SIZE

Refer to Section 5, Statistical Design and Analysis, for more details. We estimate that we will be able to enroll 1000 children who are excluded from ITIP1 and ITIP2. This estimate assumes that a quarter of the children screened for ITIP1 or ITIP2 will be ineligible or not interested in participating in the clinical trials and that 80% of those children will be eligible and willing to enroll in ITIP3. Based on the anticipated sample size for ITIP3 in conjunction with two estimates of the prevalence of the four high frequency and high mortality co-morbidities within ITIP3, we performed calculations to obtain the effect sizes that would need to be observed for comparisons between studies to achieve 80% power.

Available data from KCH and Malawi estimate that the prevalence of severe pneumonia is 10% to 24%, severe malaria is 15% to 20%, SAM is between 4% and 7%, and HIV is 5% to 10%. Based on the estimated treatment failure rates for ITIP1 and ITIP2, we conservatively estimate that clinical cure will be observed in 90% to 95% of children in ITIP1 and between 85% and 90% in ITIP2. The table below shows the effect size we would be able to see for various comparisons between ITIP3 and ITIP1 or ITIP2, along with different estimated prevalence rates. For example, comparing clinical cure among those with HIV infection or exposure in ITIP3 and those in the standard care arm of ITIP2, if the prevalence of HIV infection or

exposure is 10% in ITIP3, we will have 80% power to detect an absolute difference in proportions of 9.9% if at least 90% of the children in ITIP2 are cured and 80.1% of the children with HIV infection or exposure in ITIP3 are cured.

Observable effect sizes for ITIP3 comparisons with ITIP1 or ITIP2*

Exposure of interest	Prevalence of exposure (ITIP3)	N (ITIP1 or ITIP2)	N (ITIP3)§	Absolute observable difference	Prevalence of clinical cure (ITIP1 or ITIP2)	Prevalence of clinical cure (ITIP3)
Severe Malaria	20%	1000	200	5.5%	95%	89.5%
		1000	200	7.2%	90%	82.8%
		1000	200	8.3%	85%	76.7%
	15%	1000	150	6.3%	95%	88.7%
		1000	150	8.1%	90%	81.9%
		1000	150	9.4%	85%	75.6%
Anemia	10%	1000	100	7.6%	95%	87.4%
		1000	100	9.9%	90%	80.1%
		1000	100	11.4%	85%	73.6%
	7.5%	1000	75	8.8%	95%	86.2%
		1000	75	11.3%	90%	78.7%
		1000	75	13.1%	85%	72.0%
HIV infection or exposure	10%	1000	100	7.6%	95%	87.4%
		1000	100	9.9%	90%	80.1%
		1000	100	11.4%	85%	73.6%
	5%	1000	50	10.9%	95%	84.1%
		1000	50	13.9%	90%	81.3%
		1000	50	15.9%	85%	69.1%
SAM	7%	1000	70	9.1%	95%	85.9%
		1000	70	11.7%	90%	78.3%
		1000	70	13.5%	85%	71.5%
	4%	1000	40	12.2%	95%	82.8%
		1000	40	15.6%	90%	74.4%
		1000	40	17.8%	85%	67.2%

* Alpha set to 5% and power set to 80%.
§ Assume similar numbers for fast-breathing AND for chest-indrawing to be able to make the comparisons for the ITIP1 and ITIP2 cohorts.

Note that the absolute observable difference is largest for SAM, as that outcome is expected to have the lowest prevalence among the outcomes of interest. Nonetheless, we believe all of the estimated effect sizes to be clinically relevant. We believe that ITIP3 will have sufficient power unless the prevalence rates are very small (e.g., 4% for SAM) and the prevalence rates of clinical cure in the clinical trials are low as well (e.g., 85% cure in either ITIP1 or ITIP2).

4.6 STUDY PROCEDURES

Refer to Appendix I for Study Procedures and Visits Table. Refer to Appendix II for Laboratory Specimens Collection, Timing and Distribution Table.

Figure 1. Study Flow Chart

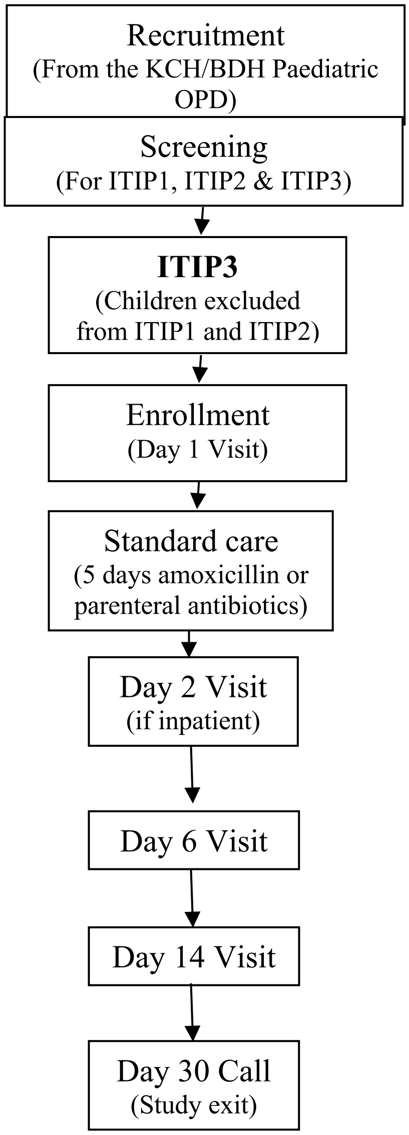

3. Recruitment

Recruitment for this study will be performed by KCH or BDH study staff during screening for ITIP1 or ITIP2. Children between 2 to 59 months of age presenting to the OPD with cough or difficult breathing will be assessed by hospital staff for potential referral to ITIP1 or ITIP2, and if meeting exclusion criteria for those studies, will be assessed by study staff for ITIP3. For any children with a cough fewer than 14 days or difficult breathing, the clinician will read to the caregiver an ITIP recruitment script (refer to Appendix V) with a brief introduction to the study. If the caregiver is interested in learning more about the study and in potentially having the child assessed for eligibility, he/she will be referred to study staff.

All KCH or BDH staff involved in recruitment procedures will be trained in relevant study-specific procedures and certified in standard Good Clinical Practices (GCP). Each recruitment and referral interaction will be documented for study records. Due to busy clinic workflow, the study may provide additional staffing assistance in the OPD, in which case initial recruitment efforts may also be performed by study staff responsible for standard KCH or BDH duties.

4. Screening

Screening procedures are conducted by study staff to simultaneously determine eligibility for enrollment in ITIP1, ITIP2, or ITIP3. All inclusion/exclusion criteria must be assessed on presentation. The following procedures are performed for screening:

- Provide information on the studies
- Obtain written informed consent for screening
- Assign participant identification (ID) number
- Collect demographic and address information

- Collect medical history
- Assess all eligibility criteria, including respiratory rate, chest-indrawing and pulse oximetry assessments (if not already documented in the medical record from that day) as well as a targeted physical examination
- Perform mRDT. Those who are found to have malaria will receive appropriate antimalarial treatment using artemisinin-based combination therapy
- Perform HIV rapid antibody testing if HIV status unknown
- Perform hemoglobin test (HemoCue®) for anemia

Note that if a child presents with wheezing (audible or auscultatory), study staff will administer a trial of rapid acting inhaled bronchodilator for up to three times, 15-20 minutes apart. Study staff will then assess for fast-breathing and chest-indrawing again to determine the child's eligibility for this study.

All screening procedures will be conducted by study staff, with the possible exception of the HIV rapid antibody test. HIV testing may be performed by either study staff or a team at KCH or BDH specially trained and experienced in pediatric HIV counseling and testing, whichever will reduce wait times for potential study participants and minimize disruption in regular care provision at KCH or BDH. Caregivers will be informed of all screening results during the screening visit, regardless of the eligibility status of their child.

For those children who are not eligible for any of the three studies, study staff will inform the caregiver(s) that their child will not be able to participate in the study and will receive standard care at KCH or BDH instead. Children less than 24 months of age or breastfeeding with an HIV-positive rapid antibody test result will be referred for confirmatory testing (e.g., dried blood spot filter paper test).

All screening procedures will be documented in the appropriate study forms, including logs and case report forms (CRFs). Clinical assessments and findings will also be documented in the child's medical record, as appropriate.

5. Informed Consent

For the purposes of this protocol, "caregiver" refers to the legally authorized representative (LAR) of the child and informed consent may only be obtained from a child's LAR. Both mother and father are considered LARs for a child, so consent may be obtained from either parent. In the absence of a biological parent, documented proof of legal guardianship would be needed to establish a caregiver's status as a LAR.

This study will have two informed consent forms (ICFs): one for screening procedures and one for enrollment procedures. The ICF used for screening is identical to that used for ITIP1 and ITIP2. Informed consent is the process of ensuring that caregivers of children fully understand what will and may happen to their children while participating in a research study. Study staff will administer a comprehension checklist to potential participants' caregivers prior to obtaining written informed consent for enrollment to ensure that caregivers fully comprehend the nature of the study. The informed consent process continues

throughout the study. Key study concepts will be reviewed periodically with the caregivers and the review will be documented. Additionally, if any new information is learned that may affect the caregiver's decision to stay in the study, this information will be shared with the caregivers in writing. All consent materials will be approved by the appropriate Institutional Review Board (IRB) and Independent Ethical Committee (IEC) prior to use.

Refer to detailed description of informed consent procedures and ethical committee approval in Section 6 (Ethical Considerations and Consent).

- **Enrollment Visit**

  After screening is complete, study staff will perform the ITIP3 enrollment visit procedures for the study for only those children who are still eligible for the observational study. For those children who are eligible for ITIP3, the following procedures are performed for enrollment:
    - Administer comprehension checklist
    - Obtain written informed consent for enrollment
    - Perform a physical exam including vital signs and an assessment of any baseline characteristics not already recorded in the medical record or assessed during screening, including measurement of MUAC
    - Collect vaccination history and additional socio-demographic information
    - Collect locator information to be able to contact caregiver and conduct a home visit, if necessary
    - Prescribe antibiotics and concomitant medications, as necessary (e.g., antimalarials if mRDT-positive) according to KCH standard care
    - Admit to the hospital, if indicated per KCH standard care

  All enrollment procedures will be documented in the appropriate study forms. Clinical assessments and findings will also be documented in the child's medical record, as appropriate.

6. Management of Study Participants During Hospitalization

All children enrolled in ITIP3 will be managed according to KCH standard care. Children in the study will be primarily managed by study clinicians during any hospitalization, including ward rounds and clinical assessments. Diagnostic tests and medication for intercurrent illnesses will be ordered per ward protocols with results documented in study files. This includes antibiotic treatment regimen changes. Study clinicians will be informed by hospital clinicians about the clinical care of children in the study and any clinical decisions made by hospital staff. Study staff will be responsible for orders in the event that study-related laboratory tests and specimen collection are required. The study will be responsible for costs incurred from any laboratory tests performed solely for study purposes. In addition to all study staff, all KCH clinicians and nurses providing care for study participants will undergo GCP and IMCI training.

7. Follow-Up Visits

Target dates for follow-up visits are calculated from Day 1, the date of enrollment. All visits must occur on the calendar day on which they are initially scheduled or within 24 hours afterwards, with the exception for the Day 14 visit which can occur either 2 days before or after Day 14, or the Day 30 phone call which can occur either 2 days before or 14 days after Day 30, and still be considered completed within the visit window.

For those children admitted to KCH, a follow-up visit will be conducted in the hospital on day 2. If their children are not hospitalized, caregivers will bring their children for follow-up visits on days 6 and 14. On day 30, study staff will initiate a phone call to ascertain the caregiver's assessment of the child's condition and symptoms.

Prior to discharge, caregivers will be instructed on how to contact the study site personnel for concerns that may arise between scheduled visits. Caregivers will receive an instruction sheet with details on the signs and symptoms that should prompt an immediate call to study staff. A study 24-hour hotline phone number will be provided to each caregiver and will be answered 24/7.

If a child is not still in the hospital, study staff will attempt to contact the caregiver by phone prior to scheduled study visits to remind them to return to the clinic at the appropriate time.

Follow-up visit procedures at scheduled in-person visits include the following:

- Review/update locator information
- Review results from prior visits
- Collect medical history since the last study visit
- Perform physical exam including respiratory rate, chest-indrawing and pulse oximetry assessments

Follow-up visit procedures at scheduled phone call include the following:

- Collect medical history since the last study visit

All follow-up visit procedures will be documented in the appropriate study forms. Clinical assessments and findings will also be documented in the child's medical record, as appropriate.

8. Missed Visits

In case of a no-show at the clinic for a scheduled in-person study visit, study personnel will call the caregiver and visit the child's home either that afternoon or the following day, to conduct the study visit. If study staff is unable to reach a caregiver by phone for the day 30 scheduled phone call, at least two repeat attempts will be made. If contact has still not been made with a caregiver, study staff will visit the child's home in order to track down the caregiver within the +14/-2 day visit window period. Maximum efforts will be made to ensure complete follow-up in the study. For children who do not complete a scheduled visit within the visit window, that visit will be documented as "missed" but study staff will still

attempt to complete the appropriate assessments from that visit, if possible (e.g., Day 6 visit performed and documented on Day 9).

Based on our current experience, we expect that fewer than 5% of the children will be lost to follow-up at the time of primary outcome assessment.

9. Interim Contacts and Visits

Interim contacts and visits (those between regularly scheduled follow up visits) may be performed at caregiver request or as deemed necessary by the site investigators or designee at any time during the study. All interim contacts and visits will be documented in the child's study records and on applicable CRFs. Interim visits may occur at the study clinic or at the child's home.

Study staff will encourage caregivers to call the 24/7 study hotline if they observe any symptoms of concern in their child.

10. Withdrawal and Early Termination

Children and their caregivers may voluntarily withdraw from the study for any reason at any time. Any participant withdrawal or early termination will be documented in the appropriate study forms. Any child withdrawn from the study will continue to receive local standard of care.

11. Study Termination Visit

The Day 30 phone call will serve as the study termination visit for the study.

12. Biohazard Containment

As exposure to blood-borne pathogens can occur through contact with contaminated needles, blood, and blood products, appropriate blood and secretion precautions will be employed by all personnel in the HIV, anemia, and malaria testing for this study as recommended by the U.S. Centers for Disease Control. Biohazardous waste will be contained according to institutional, transportation/carrier, and all other applicable regulations.

4.7 STUDY MEDICATION USE

Standard of care per Malawian guidelines and/or KCH ward protocol will be provided (e.g., 5 days of oral amoxicillin or, for severe pneumonia, parenteral ampicillin/ penicillin and gentamicin).

Caregivers will be given the emergency contact number for the study personnel during the consenting process in order to report any AEs.

4.8 DATA COLLECTION

Clinical research data will be maintained through a combination of secure electronic data management system and physical files with restricted access. Data related to study endpoints will be extracted from the electronic databases for statistical analysis. Three distinct study databases will be created and maintained: the primary study database with study visit data, a safety database with serious adverse event (SAE) assessments, and a database with participating children's personally identifiable information. The first two study databases containing study endpoint data will identify children only by study identification numbers and will not contain identifying information such as name, address, medical record number or personal contact information. In the third database, the study coordinator will maintain a log that will contain the link between personal identifiers and the study participant IDs. The linklog and any other documentation (paper-based or electronic) that has both personal identifiers and the participant ID will have restricted access and will be stored in a secure manner separately from other study data and will be retained for at least five years after the last participating child exits the study.

13. Case Report Forms

All study data will be collected by the clinical study staff using designated source documents or paper-based CRFs. Study data will be entered directly into the CRFs during a study visit. Data from the paper-based CRFs will be entered after the fact into the electronic database as promptly as is feasible. Study staff will maintain source documents for each child at the study site. Source documentation will be available for review to ensure that the collected data are consistent with the CRFs. CRFs and laboratory reports will be reviewed by the site clinical team who are responsible for ensuring that they are accurate and complete. CRFs, source documents and other supporting documents (both electronic and paper-based) will be kept in a secure location and remain separate from participant identification information (name, address, etc.) to ensure confidentiality.

14. Source Documents

Source documents include but are not limited to:

- Signed informed consent forms
- Documentation of the comprehension checklist
- Visit documentation that includes dates of study visits
- Receipts for travel reimbursement
- Reported laboratory results
- Clinic notes
- Prescription records

A copy of all laboratory results will also be included in the child's medical records. Site investigators will maintain, and store in a secure manner, all source documents throughout the study. These documents will be retained for at least five years after the last child exits the study.

4.9 DATA MANAGEMENT

Primary data management activities will be undertaken by the designated contract research organization (CRO). The on-site study data manager will oversee data-related procedures at the study site and will be supervised by the CRO data management staff. Data management activities include data entry and validation, data coding and cleaning, database quality control, disaster recovery plans, preparation and submission of reports to the Sponsor, and preparation of final study database. Data management activities will be performed using Clindex® Clinical Trial and Data Management software, developed by Fortress Medical Systems.

15. Data Access

The participating site will maintain appropriate medical and research records for this study, in compliance with International Conference on Harmonization Good Clinical Practice E6 (ICH-GCP), regulatory, sponsoring organization and institutional requirements for the protection of confidentiality of children. The site will permit authorized representatives of the Sponsor and regulatory agencies to examine (and when required by applicable law, to copy) clinical records for the purposes of quality assurance reviews, audits and evaluation of the study safety and progress. User-specific usernames and passwords are required to log onto the database. User rights will be provided to study staff, PIs, and co-investigators at the level appropriate for each individual's job description.

- **Data Storage**

The site investigators and designees will maintain, and store securely, complete, accurate and current study records throughout the study. In accordance with regulations, study staff will retain all study records on site for at least five years after study closure. Study records will not be destroyed prior to receiving approval for record destruction from the Sponsor. Applicable records include source documents, site registration documents and reports, informed consent forms, and notations of all contacts with the child.

The Clindex® database is hosted by Fortress Medical Systems through their Software as a Service platform and accessed remotely online. All of the servers that host the Clindex® software and data are housed at ATOMICdata, a Tier 3, SOC 3 Certified Data Center. The primary hosting facility is at the ATOMICdata Minneapolis South facility.

16. External Study Monitoring

The Study Sponsor is responsible for contacting and visiting the study site for the purpose of inspecting the facilities and, upon request, inspecting the various records of the study. Participant confidentiality will be respected.

4.10 ASSESSMENTS AND REPORTING

17. Monitoring

The study site investigators will be responsible for close safety monitoring of all children participating in the study, and for alerting the protocol team if unexpected concerns arise. All

children will undergo a targeted physical exam at screening and enrollment to ensure that children are medically stable and do not demonstrate any exclusion criteria. Each participating child will be evaluated by a study clinician at each in-person study visit. If a child misses an in-person study visit, home visits will be conducted by trained study staff to ensure clinical evaluation. Every effort will be made to trace all children in the study for the final outcome assessment.

18. Adverse Events

All AEs will be managed by the clinical study site team in accordance with the standard clinical practices in place at the hospital. The clinical team will assess and treat or refer the participating child for medical care as appropriate, which may include additional study visits, if necessary.

The protocol team anticipates AEs, both severe and non-severe, to occur among enrolled children at a similar rate as untoward medical events occur in comparable pediatric populations outside of a research setting. AEs that the study team expects may occur during the research include, but are not limited to: adverse reactions to amoxicillin (e.g., skin rash), onset of pneumonia-related symptoms (e.g., fever), and onset of other common and uncommon childhood illnesses (e.g., diarrhea, measles).

19. Serious Adverse Event

SAEs will be defined as AEs occurring that:

a. Result in death
b. Are life-threatening AEs
c. Require inpatient hospitalization or prolongation of existing hospitalization
d. Result in persistent or significant disability/incapacity, or
e. Are congenital anomalies/birth defects.

Important medical events that may not result in death, be life-threatening, or require hospitalization may be considered serious when, based upon appropriate medical judgment, they may jeopardize the health of the participating child or require medical or surgical intervention to prevent one of the outcomes listed above.

Note that the initial hospitalization for any child admitted at enrollment does not count as an SAE as the condition for which the child was hospitalized occurred prior to enrollment in the study, classifying it as a pre-existing condition and not an AE.

The collection of data regarding AEs and SAEs in this observational study facilitates comparison of outcomes for this cohort with the ITIP1 and ITIP2 cohorts.

20. Study Discontinuation

The study may be discontinued at any time by the protocol team, Sponsor, funding agency, Malawi regulatory authorities, or institutional review board/ethics committee.

5 STATISTICAL DESIGN AND ANALYSIS

5.1 DATA ANALYSIS

21. Overview and General Design

In brief, we plan to conduct a facility-based, prospective, observational study of clinical outcomes among children 2 to 59 months of age with childhood pneumonia and other co-morbidities presenting to KCH or BDH in Lilongwe, Malawi who have been excluded from pneumonia clinical trials ITIP1 and ITIP2 and meet inclusion criteria for ITIP3. The study will enroll 1000 children 2 to 59 months of age with pneumonia presenting to KCH or BDH in Lilongwe, Malawi and excluded from ITIP1 and ITIP2. Each child will receive standard of care per Malawian guidelines and/or KCH ward protocol (e.g., 5 days of oral amoxicillin or, for severe pneumonia, parenteral ampicillin/ penicillin and gentamicin) and will be followed for 14 days with regular study visits at days 2 (if hospitalized as per KCH standard of care), 6, 14 and 30 (by phone) after enrollment.

22. Objectives and Endpoints

We hypothesize (stated under the alternative):

Comparisons between ITIP3 and ITIP1

a) the percentage of children who are clinically cured by day 14 will be lower among children with fast-breathing pneumonia and severe malaria in ITIP3 than among children in the standard care arm of ITIP1;

b) the percentage of children who are clinically cured by day 14 is lower among children with fast-breathing pneumonia and severe acute malnutrition (SAM) in ITIP3 than among children in the standard care arm of ITIP1;

c) the percentage of children who are clinically cured by day 14 will be lower among children with fast-breathing pneumonia and HIV in ITIP3 than among children in the standard care arm of ITIP1;

d) the percentage of children who are clinically cured by day 14 will be lower among children with fast-breathing pneumonia and anemia in ITIP3 than among children in the standard care arm of ITIP1;

Comparisons between ITIP3 and ITIP2

e) the percentage of children who are clinically cured by day 14 will be lower among children with chest-indrawing pneumonia and severe malaria in ITIP3 than among children in the standard care arm of ITIP2;

f) the percentage of children who are clinically cured by day 14 will be lower among children with chest-indrawing pneumonia and SAM in ITIP3 than among children in the standard care arm of ITIP2; and

g) the percentage of children who are clinically cured by day 14 will be lower among children with chest-indrawing pneumonia and HIV in ITIP3 than among children in the standard care arm of ITIP2.

h) the percentage of children who are clinically cured by day 14 will be lower among children with chest-indrawing pneumonia and anemia in ITIP3 than among children in the standard care arm of ITIP2;

We will also conduct exploratory investigations on whether there is a differential treatment response by gender, age, weight, MUAC, HIV-status, malaria, and vaccination status among children enrolled in ITIP3.

- **Analytical Methodology**

Generalized linear models with robust standard errors will be used to compare the percentages of children who are clinical cured by day 14 among ITIP3 children and children in the standard of care arm of ITIP1 (or ITIP2) who have severe malaria (or are HIV positive or exposed or present with SAM) while adjusting for age and vaccination status. Tests will be performed as two-sided tests with alpha = 0.05. There will be no adjustments for multiple comparisons.
Similar linear or generalized linear models will be used for secondary outcome analyses.

We anticipate that some children may not return for their scheduled follow-up visits. In addition to appointment reminders and counseling caregivers on the importance of completing follow-up, study staff will provide incentives and transportation costs to minimize missing outcome data. For those children who do not return for their scheduled follow-up in-person visits, study staff will conduct home visits the next day to assess the outcome. We have estimated the loss to follow-up to be 5% in this study.

5.2 RESULT PRESENTATION

The results of this research will be primarily presented through at least one published manuscript with detailed description of the background, methods, results, and conclusion. The specific format and details of this manuscript will be in accordance with the requirements of the publishing journal, but is expected to include tables describing the baseline characteristics of study participants for each study endpoint.

5.3 DISSEMINATION OF RESULTS

The results of this study will be published collaboratively by investigators at SCUS, UNC, UW, Malawi COM, and Malawi MOH in peer-reviewed journals. Study findings will be presented to the Malawi MOH Senior Management and hospital staff at the study site. Co-investigators plan on attending at least one international conference to disseminate the findings of the study.

6 ETHICAL CONSIDERATIONS AND CONSENT

- **Principles for Clinical Research**

  This clinical study will be conducted in compliance with the protocol and all applicable IRB/IEC reviews.

- **Institutional Review Boards (IRBs) and Independent Ethics Committees (IECs)**

  The IRB and IEC of record for this clinical study are the Western Institutional Review Board (WIRB) and the University of Malawi COM Research and Ethics Committee (COMREC). A copy of the protocol, proposed informed consent forms, other written participant information, and any proposed advertising material will be submitted to both WIRB and COMREC for written approval. The investigators must submit and, where necessary, obtain approval from the IRB/IEC for any major protocol amendments and changes to the informed consent document. The Sponsor (SCUS), CRO (Triclinium), and study operations partner (UNC) are responsible for assuring that this protocol and the associated informed consent documents and study-related documents are approved by WIRB and COMREC prior to implementation of the protocol. Any major amendments to the protocol, informed consents, or other study-related documents must be approved by the IRB/IEC prior to implementation.

- **Informed Consent**

  In obtaining and documenting informed consent, the site investigators and their designees will comply with applicable local and domestic regulatory requirements. This clinical study will have an ICF for screening and an ICF for enrollment developed for local use that are in accordance with all applicable regulations. Both an English and Chichewa version of the ICFs will be reviewed and approved by the IRB/IEC of record before use with participants. The consent forms will include the purpose of the study, a description of the procedures to be followed and the risks and benefits of participation. The informed consent process will give individuals all of the relevant information they need to decide whether to participate, or to continue participation, in this study. Potential research participants' caregivers will be encouraged to ask questions and to exchange information freely with the study team. If the caregiver providing consent is illiterate, an independent witness will be present to verify to the caregiver that all the information read aloud is contained in the ICF. In this instance, both the caregiver and witness will sign the ICF.

  Before a child begins participation in the study, it is the site investigators' responsibility to ensure that informed consent is obtained from a LAR after adequate explanation of the aims, methods, and potential risks and benefits of the study. The study staff obtaining consent will also sign and date the ICF. A signed and dated copy of the consent form will be given to the participant's caregiver and this will be documented in the child's health passport.

- **Risks to Participants**

  - Coercion

Caregivers may feel coerced to enroll in the study in order to receive care for their child within a research setting, which may be perceived as of a higher quality than the standard of care.

- Specimen Collection
  The study involves blood specimen sampling at screening and, in the presence of a fever, at outcome assessment. This is standard of care. Phlebotomy can cause pain and bruising at or around the blood draw site.

- Medical Management
  Participation in the study has the potential to compromise care for hospitalized children, if study procedures are prioritized above urgent clinical care for acute infections.

- **Protection against Risks**

  - Coercion
    In order to minimize the risk of coercion, OPD clinicians will inform caregivers about the study and refer only those who are interested. During the informed consent process, study staff will emphasize that the child will receive medical care whether enrolled in the study or not.

  - Specimen Collection
    In order to minimizing the risks associated with phlebotomy, all study staff who will be collecting specimens from children in the study will be trained in the appropriate procedures and supervised accordingly. This is part of standard of care at KCH.

  - Medical Management
    In order to minimize the possibility that participation in this study will interfere with the medical management of children with pneumonia at KCH, study staff will have the primary responsibility for the clinical management of hospitalized children. Hospitalized children (e.g., those under 6 months of age during the initial overnight admission) will be treated and managed by study staff in accordance with standard procedures. Study staff will be informed about any decisions regarding changing treatment regimens made by KCH staff. The study is prepared to hire additional staff as necessary to avoid overburdening the KCH system. Please refer to Section 4.6 Study Procedures for further description of Management of Study Participants During Hospitalization.

- **Benefits to Participants**

  Direct benefits to children in this study include increased clinical supervision and care during the study period as compared to alternatives not in a study setting. Frequent follow-up visits are not included as standard of care, so participating children will benefit from monitoring for two weeks from the pneumonia episode, including phone calls and home visits for missed follow-up. This level of supervision will make it more likely that deterioration is identified and managed accordingly as compared to in a non-study setting. Additionally, participants'

caregivers will have access to a 24/7 hotline, answered by trained staff, which is not a part of standard of care.

- **Participant Confidentiality**

  The site investigators must ensure that the child's confidentiality is maintained. Personal identifiers will not be included in any study reports. All study records will be kept confidential to the extent provided by national and local laws.

  All study procedures will be conducted to protect participant privacy and confidentiality to the fullest extent possible. The study site will establish a standard operating procedure (SOP) for confidentiality protection that includes both clinic and home visits and reflects the input of study staff and community representatives to identify potential confidentiality issues and strategies to address them.

- **Participant Reimbursement**

  Travel reimbursement will be provided to caregivers to compensate them for the cost of transport for study visits. Reimbursement will be provided in the local currency equivalent to US$5 for each scheduled or interim study visit, payable at the end of the visit. The study consent form will list the amount to be paid in the local currency. Participants' caregivers will not receive reimbursement for visits that occur while the child is hospitalized to avoid disruptions in the hospital wards with other non-study patients.

  Participants' caregivers will receive a phone card with airtime worth MK 100 on the carrier of their choice (either AirTel or TNM) to cover any phone calls the caregiver may need to make to study staff during the course of the study.

  Study participants' caregivers will not be responsible for paying for study-related drugs, tests, or examinations.

- **Storage of Specimens**

  Specimens collected during the course of this research will not be stored. Any leftover samples not consumed during study-related diagnostic tests will be destroyed.

7 POSSIBLE CONSTRAINTS

Anticipated implementation challenges to the successful outcome of the study include:

1. Ensuring quality and consistency of implementation at the study site. We plan to provide standardized training, supervision, and oversight to ensure quality and harmonized study procedures. A CRO will be contracted to provide additional oversight and monitoring of the site, as needed.
2. Following up all children. Recognizing that some children may not come back for the follow-up visits, we plan to include and train study staff to locate children who miss their follow-up

appointments and conduct these visits in the home. We will also ensure that study staff take the time to educate caregivers on the importance of adhering to the treatment regimen and follow-up.

8 REQUIREMENTS AND TRAINING

See Appendix VII for a description of study requirements for study activities, including training for study personnel and KCH/BDH staff.

9 BUDGET

- **Budgetary Estimates**

The program of activities to perform the work outlined in this protocol includes the cost of the sub-awards as all other costs (personnel and fringe benefits, travel, consultants, supplies, and indirect costs) are part of the overall ITIP project budget, and are already included in the concurrent ITIP1 and ITIP2 study budgets. No additional Save the Children personnel and fringe benefits, travel costs, direct supplies and other are budgeted to carry out this third study. The additional expenses required from the funder, the Bill & Melinda Gates Foundation (BMGF), are for the sub-grants to University of North Carolina Lilongwe Trust for study implementation and Triclinium for study monitoring. See Table 5 below for more detail.

Table 5. ITIP3 Initial Budget Estimates

**Budget Category**	**Budget (in $ US Dollars)**			
	**Year 1**	**Year 2**	**Year 3**	**Total**
Total Direct FTEs	-	-	-	-
Total Direct Travel	-	-	-	-
Total Direct Supplies	-	-	-	-
Sub-grant University of North Carolina				
Nurse (50%)	2,325	4,650	2,325	
Data associate (50%)	1,373	2,745	1,373	
Administrator (10%)	536	1,073	536	
Indirect staffing costs (12.5%)	1,401	2,802	1,401	
Reimbursement (transportation costs)	6,500	7,000	6,500	
Airtime	150	200	150	
Study specific tests	2,300	2,800	2,300	
Total	14,585	21,270	14,585	50,440
Sub-grant University of Washington	-	-	-	-
Sub-grant Triclinium				
Data management	2,800	2,940	2,646	
Monitoring activities	1,200	1,260	1,764	
Total	4,000	4,200	4,410	12,610
TOTAL DIRECT COSTS	20,000	21,000	22,050	63,050
TOTAL INDIRECT COSTS (outside of	-	-	-	-

Malawi)				
**GRAND TOTAL COSTS**	20,000	21,000	20,050	63,050

- **Budget Justification**

Subcontracts: SCUS/SCI collaborators are fundamental to the success of the proposal. These partners were carefully selected based on qualification factors and the ability to help SCUS/SCI achieve its objectives. SCUS/SCI has extensive experience in selecting, evaluating, and working with other organizations to achieve project objectives. Project staff, supported by SCUS/SCI Grants and Contract Services team and Legal Services team, are well versed in conducting due diligence when selecting prospective partners and in setting up appropriate contractual mechanisms for managing the relationships, accounting for grant funds in a responsible manner, and ensuring that project milestones are achieved with the funds available. SC ensures that sub-grantee direct and indirect costs conform to the budget guidelines set by the BMGF.

The proposed sub-awards budget includes the following subcontracts for this project and include a 5% inflation factor for years 2 and 3:

- University of North Carolina (UNC) Project Lilongwe Trust. The budget is to support the engagement of additional ITIP Project UNC study staff as well as to provide primary study support in Lilongwe, Malawi. Study support includes study personnel for enrollment of children and data entry/management/cleaning as well as direct study costs such as transportation reimbursements, airtime and study-specific tests. The salaries of LPIs, Drs. Ajib Phiri and Tisungane Mvalo are covered by the overall ITIP project budget, and are already included in the concurrent ITIP1 and ITIP2 study budgets.
- Triclinium Clinical Trials Project Management (Pty) Ltd. The budget is reserved for the engagement of a contract research monitor group to provide additional data management activities and objective monitoring and observation of the study as it is conducted in Malawi.

10 REFERENCES

1. World Health Organization. (2014). *Integrated management of childhood illness: chart booklet*. Geneva: World Health Organization.
2. Hazir T, Y. N. (2006). Chest radiography in children aged 2-59 months diagnosed with non-severe pneumonia as defined by World Health Organization: descriptive multi-centre study in Pakistan. *BMJ, 333*(7569), 629.
3. Hazir T, Y. N. (2011). Comparison of oral amoxicillin with placebo for the treatment of World Health Organization-defined nonsevere pneumonia in children aged 2-59 months: a multicentre, double-blind, randomized, placebo-controlled trial in Pakistan. *Clinical Infectious Diseases, 52*(3), 293-300.
4. Mulholland EK, E. S. (1992). Standardized diagnosis of pneumonia in developing countries. *Pediatric Infectious Disease Journal, 11*, 77-81.

5. World Health Organization. (2013). *Guidelines for the management of common illnesses with limited resources.* Geneva: World Health Organization.
6. English M, Scott JA. What is the future for global case management guidelines for common childhood diseases? *PLoS Medicine.* 2008;5:e241-e241.
7. K, M. (2014). New Diagnostics for Common Childhood Infections. *New England Journal of Medicine, 370*, 875-877.
8. World Health Organization. (2014). *Integrated management of childhood illness: chart booklet*. Geneva: World Health Organization.
9. Grant GB, C. H. (2009). Recommendations for treatment of childhood non-severe pneumonia. *Lancet Infect Dis, 9*, 185-196.
10. Kabra SK, L. R. (2010). Antibiotics for community-acquired pneumonia in children. *Cochrane Database of Systematic Reviews* (3).
11. World Health Organization. (2012). *Recommendations for management of common childhood conditions: evidence for technical update of pocket book recommendations: newborn conditions, dysentery, pneumonia, oxygen use and delivery, common causes of fever, severe acute malnutrition and supportive care.* Geneva: World Health Organization.
12. World Health Organization. (2003 Sep 29-Oct 1). *Consultatitive Meeting to Review Evidence and Research Priorities in the Management of Acute Respiratory Infections (ARI).* Meeting Report, Chapter: "Non-severe pneumonia: ARI Case Management: Is Antimicrobial Therapy Necessary?." Geneva: World Health Organization.
13. Thea DM, Vy N, Tran AT, Thula S, Qazi S, Fontaine O, and the NARIMA Study Group. A multicentre observational study of clinical outcome following amoxicillin treatment of non-severe acute respiratory tract infection (NARIMA). Global Congress on Lung Health, 32nd World Conference on International Union against Tuberculosis and Lung Disease (IUATLD), Paris, France, 1-4 November 2001. *Int J Tub and Lung Dis* 2001; 5 (Suppl 1):S11.
14. Thea, Donald. A multi-center prospective observational study of clinical outcome following amoxicillin treatment of non-severe acute respiratory tract infection. (2001). Unpublished final report.
15. Hopstaken RM, Witbraad T, van Engelshoven JMA, et al. Inter-observer variation in the interpretation of chest radiographs for pneumonia in community acquired lower respiratory tract infections. *Clin Radiol* 2004; **59 :** 743–52.
16. Davies HD, Wang EE, Manson D, et al. Reliability of the chest radiograph in the diagnosis of lower respiratory infections in young children. *Pediatr Infect Dis J* 1996; **15:** 600–04.
17. Sadruddin S. (2014). Save the Children. Toronto. *Personal communication.*
18. Bradley JS, et al. (2011). The management of community-acquired pneumonia in infants and children older than 3 months of age: clinical practice guidelines by the Pediatric Infectious Diseases Society and the Infectious Diseases Society of America. *Clin Infect Dis*, 53(7): e25-76.
19. Rambaud-Althaus, C, et al. (2015). Clinical features for diagnosis of pneumonia in children younger than 5 years: a systematic review and meta-analysis. Lancet Infect Dis, 15: 439-500.
20. Weerakkody Y, Paks M, et al. Lobar pneumonia. *Radiopaedia*. Accessed March 6, 2015. http://radiopaedia.org/articles/lobar-pneumonia
21. Muro F, Mtove G, Mosha N, Wangai H, Harrison N, et al. (2015). Effect of context on respiratory rate measurement in identifying non-severe pneumonia in African children. *Trop Med Int Health*, doi: 10.1111/tmi.12492

22. D'Acremont V, K. M. (2014 Feb 27). Beyond malaria--causes of fever in outpatient Tanzanian children. *New England Journal of Medicine, 370*(9), 809-17.
23. Hazir T, S. Q. (2004). Assessment and management of children aged 1-59 months presenting with wheeze, fast breathing, and/or lower chest indrawing; results of a multicentre descriptive study in Pakistan. *Archives of Disease in Childhood, 89*(11), 1049.
24. Lochindarat S, S. Q. (2008). Are we adequately managing children with wheeze using the standard case management guidelines? *Journal of the Medical Association of Thailand, 91*(Suppl 3), 60-8.
25. Lassi ZS, R. K. (2014). Antibiotic therapy versus no antibiotic therapy for children aged two to 59 months with WHO-defined non-severe pneumonia and wheeze. *Cochrane Database of Systematic Reviews,* (5).
26. Spurling GKP, J. D. (2011). Antibiotics for bronchiolitis in children. *Cochrane Database of Systematic Reviews,* (6).
27. Hazir T, N. Y. (2011 Feb 1). Comparison of oral amoxicillin with placebo for the treatment of world health organization-defined nonsevere pneumonia in children aged 2-59 months: a multicenter, double-blind, randomized, placebo-controlled trial in Pakistan. *Clinical Infectious Diseases, 52*(3), 293-300.
28. Low DE, P. M. (2004 Mar). Optimizing antibacterial therapy for community-acquired respiratory tract infectionsin children in an era of bacterial resistance. *Clin Pediatr (Phila), 43*(2), 135-51.
29. Ginsburg AS, L. T. (2013 Dec). Antibiotic non-susceptibility among Streptococcus pneumoniae and Haemophilus influenzae isolates identified in African cohorts: a meta-analysis of three decades of published studies. *Int J Antimicrob Agents, 42*(6), 482-91.
30. Neu HC. (1992). The crisis in antibiotic resistance. *Science, 257*(5073), 1064-1073.
31. Makoka MH, Miller WC, Hoffman IF, et al. (2012). Bacterial infections in Lilongwe, Malawi: aetiology and antibiotic resistance. BMC Infectious Diseases. 12:67.
32. Cornick JE, Harris SR, Perry CM, et al.(2014). Genomic identification of a novel co-trimoxazole resistance genotype and its prevalence amongst Streptococcus pneumonia in Malawi. J Antimicrob Chemother. 69(2):368-74.
33. UN Commission on Life-Saving Commodities for Women and Children. (2012 Sep). *Commissioner's Report.* New York: UN.
34. Rizal A, J. B. (2010). Acute respiratory infection: Boston university's collaborative research work in the last decade. *Indian Pediatrics 47*(1), 19-24.
35. Hussain, H., H. Waters, et al. (2006). "The cost of treatment for child pneumonias and meningitis in the Northern Areas of Pakistan." Int J Health Plann Manage **21**(3): 229-38.
36. Edejer, T. T., M. Aikins, et al. (2005). "Cost effectiveness analysis of strategies for child health in developing countries." BMJ **331**(7526): 1177.
37. Side Effects: List amoxicillin side effects by likelihood and severity. *WebMD*. Accessed March 2, 2015. http://www.webmd.com/drugs/2/drug-1531-3295/amoxicillin-oral/amoxicillin-oral/details/list-sideeffects
38. Peders J, et al. (2006). Factors influencing the composition of the intestinal microbiota in early infancy. *Pediatrics*, 118(2): 511-21.
39. Buffie CG, et al. (2011). Profound alterations of intestinal microbiota following a single dose of clindamycin results in sustained susceptibility to Clostridium difficile-induced colitis. *Infect Immun*, 80(1): 62-73.

40. Bailey MT. (2012). The contributing role of the intestinal microbiota in stressor-induced incrased in susceptibility to enteric infection and systemic immunomodulation. *Horm Behav*, 62(3): 286-94.
41. Prasad V, et al. (2013). A decade of reversal: an analysis of 146 contradicted medical practices. *Mayo Clin Proc*, 88(8): 790-798.
42. Venekamp RP, Sanders SL, Glasziou PP, et al. (2015). Antibiotics for acute otitis media in children. Cochrane Database of Systematic Reviews, (6).
43. Alves Galvao MG, et al. (2014). Antibiotics for preventing suppurative complications from undifferentiated acute respiratory infections in children under five years of age. *Cochrane Database of Systematic Reviews* (2).
44. United Nations Development Programme (UNDP). (2014). *Country Profiles, Malawi.* UNDP.
45. F, D. (2009). Epidemiology of Malaria in Malawi. In B. C. Geubbels E, *The Epidemiology of Malawi.* University of Malawi: Division of Community Health, College of Medicine.
46. United Nations AIDS Program (UNAIDS). (2014). *Country Progress Reports, Malawi.* UNAIDS.
47. World Medical Association (WMA). (October 2013). *Declaration of Helsinki: Ethical Principles for Medical Research Involving Human Subjects*. Fortaleza, Brazil: WMA General Assembly.
48. Department of Health, Education, and Welfare. (April 18, 1979). *Ethical Principles and Guidelines for the Protection of Human Subjects of Research*. Washington, DC: The National Commission for the Protection of Human Subjects of Biomedical and Behavioral Research.

APPENDICES

APPENDIX I: SCHEDULE OF STUDY VISITS AND EVALUATIONS

	Screening	Enrollment (Day 1)	Day 2	Day 6	Day 14	Interim visit(s)	Day 30
Informed Consent	✓	✓					
Comprehension Checklist		✓					
Participant ID	✓	✓	✓	✓	✓	✓	✓
Eligibility Assessment	✓	✓					
Demographics	✓						
Locator Information	✓	✓	✓	✓	✓	✓	
Reimbursement		✓	✓	✓	✓	✓	
Schedule Next Visit	✓	✓	✓	✓			
Medical History	✓	✓	✓	✓	✓	✓	✓
Targeted Physical Exam	✓	✓	✓	✓	✓	✓	

APPENDIX II: SAMPLE COLLECTION AND LABORATORY EVALUATIONS

Specimen for Diagnostic	Screening	Enrollment (Day 1)	Day 2	Day 6	Day 14	Day 30	Labor-atory
HIV test	✓						Study site
Anemia test	✓						
Malaria test	✓						

APPENDIX III: SAMPLE SIZE CALCULATIONS

Hypotheses below are all stated under the alternative.

Hypothesis 1:

Comparisons between ITIP3 and ITIP1

The percent of children who are cured by day 14 will be lower among children who have severe malaria and fast-breathing pneumonia who are excluded from the trials than among children in the standard care arm in the ITIP1 trial.

We present estimates of effect size (column "delta") (difference in percent of children who are cured by day 14 under the assumption that the ITIP3 will enroll 1000 children and that either 15% or 20% have severe malaria with a two-sided test of proportions, an alpha level of 0.05 and 80% or 90% power.

Prevalence of severe malaria of 15% and percent of outcome of 95% and 90% (column p1) in the ITIP1 (referent) group:

alpha	Power	N	N1	N2	delta	p1	p2
0.05	80%<br>90%	1150	1000	150	-0.063<br>-0.077	0.95<br>0.95	0.89<br>0.87
0.05	80%<br>90%	1150	1000	150	-0.081<br>-0.098	0.90<br>0.90	0.82<br>0.80

Prevalence of severe malaria of 20% and percent of outcome of 95% and 90% (column p1) in the ITIP1 (referent) group:

alpha	Power	N	N1	N2	delta	p1	p2
0.05	80%<br>90%	1200	1000	200	-0.055<br>-0.067	0.95<br>0.95	0.90<br>0.88
0.05	80%<br>90%	1200	1000	200	-0.072<br>-0.086	0.90<br>0.90	0.83<br>0.81

Hypothesis 2:

Comparisons between ITIP3 and ITIP1

The percent of children who are cured by day 14 will be lower among children who have SAM and fast-breathing pneumonia who are excluded from the trials than among children in the standard care arm in the ITIP1 trial.

We present estimates of effect size (column "delta") (difference in percent of children who are cured by day 14 under the assumption that the ITIP3 will enroll 1000 children and that either 4%

or 7% have SAM with a two-sided test of proportions, an alpha level of 0.05 and 80% or 90% power.

Prevalence of SAM of 4% and percent of outcome of 95% and 90% (column p1) in the ITIP1 (referent) group:

alpha	Power	N	N1	N2	delta	p1	p2
0.05	80%<br>90%	1040	1000	40	-0.122<br>-0.155	0.95<br>0.95	0.83<br>0.80
0.05	80%<br>90%	1040	1000	40	-0.156<br>-0.191	0.90<br>0.90	0.74<br>0.71

Prevalence of SAM of 7% and percent of outcome of 95% and 90% (column p1) in the ITIP1 (referent) group:

alpha	Power	N	N1	N2	delta	p1	p2
0.05	80%<br>90%	1070	1000	70	-0.091<br>-0.114	0.95<br>0.95	0.86<br>0.84
0.05	80%<br>90%	1070	1000	70	-0.117<br>-0.142	0.90<br>0.90	0.78<br>0.76

Hypothesis 3:

Comparisons between ITIP3 and ITIP1

The percent of children who are cured by day 14 will be lower among children who have HIV and fast-breathing pneumonia who are excluded from the trials than among children in the standard care arm in the ITIP1 trial.

We present estimates of effect size (column "delta") (difference in percent of children who are cured by day 14) under the assumption that the ITIP3 will enroll 1000 children and that either 5% or 10% have HIV with a two-sided test of proportions, an alpha level of 0.05 and 80% or 90% power.

Prevalence of HIV of 5% and percent of outcome of 95% and 90% (column p1) in the ITIP1 (referent) group:

alpha	Power	N	N1	N2	delta	p1	p2
0.05	80%<br>90%	1050	1000	50	-0.109<br>-0.137	0.95<br>0.95	0.84<br>0.81
0.05	80%<br>90%	1050	1000	50	-0.139<br>-0.170	0.90<br>0.90	0.76<br>0.73

Prevalence of HIV of 10% and percent of outcome of 95% and 90% (column p1) in the ITIP1 (referent) group:

alpha	Power	N	N1	N2	delta	p1	p2
0.05	80%<br>90%	1100	1000	100	-0.076<br>-0.094	0.95<br>0.95	0.87<br>0.86
0.05	80%<br>90%	1100	1000	100	-0.099<br>-0.119	0.90<br>0.90	0.80<br>0.78

Hypothesis 4:

Comparisons between ITIP3 and ITIP2

The percent of children who are cured by day 14 will be lower among children who have severe malaria and chest-indrawing pneumonia who are excluded from the trials than among children in the standard care arm in the ITIP2 trial.

We present estimates of effect size (column "delta") (difference in percent of children who are cured by day 14 under the assumption that the ITIP3 will enroll 1000 children and that either 15% or 20% have severe malaria with a two-sided test of proportions, an alpha level of 0.05 and 80% or 90% power.

Prevalence of severe malaria of 15% and percent of outcome of 90% and 85% (column p1) in the ITIP2 (referent) group:

alpha	Power	N	N1	N2	delta	p1	p2
0.05	80%<br>90%	1150	1000	150	-0.081<br>-0.098	0.90<br>0.90	0.82<br>0.80
0.05	80%<br>90%	1150	1000	150	-0.094<br>-0.112	0.85<br>0.85	0.76<br>0.74

Prevalence of severe malaria of 20% and percent of outcome of 90% and 85% (column p1) in the ITIP2 (referent) group:

alpha	Power	N	N1	N2	delta	p1	p2
0.05	80%<br>90%	1200	1000	200	-0.072<br>-0.086	0.90<br>0.90	0.83<br>0.81
0.05	80%<br>90%	1200	1000	200	-0.083<br>-0.098	0.85<br>0.85	0.77<br>0.75

Hypothesis 5:

Comparisons between ITIP3 and ITIP2

The percent of children who are cured by day 14 will be lower among children who have SAM and chest-indrawing pneumonia who are excluded from the trials than among children in the standard care arm in the ITIP2 trial.

We present estimates of effect size (column “delta”) (difference in percent of children who are cured by day 14 under the assumption that the ITIP3 will enroll 1000 children and that either 4% or 7% have SAM with a two-sided test of proportions, an alpha level of 0.05 and 80% or 90% power.

Prevalence of SAM of 4% and percent of outcome of 90%, 85% and 80% (column p1) in the ITIP2 (referent) group:

alpha	Power	N	N1	N2	delta	p1	p2
0.05	80%<br>90%	1040	1000	40	-0.156<br>-0.191	0.90<br>0.90	0.74<br>0.71
0.05	80%<br>90%	1040	1000	40	-0.178<br>-0.214	0.85<br>0.85	0.67<br>0.64
0.05	80%<br>90%	1040	1000	40	-0.194<br>-0.230	0.80<br>0.80	0.61<br>0.57

Prevalence of SAM of 7% and percent of outcome of 90%, 85% and 80% (column p1) in the ITIP2 (referent) group:

alpha	Power	N	N1	N2	delta	p1	p2
0.05	80%<br>90%	1070	1000	70	-0.117<br>-0.142	0.90<br>0.90	0.78<br>0.76
0.05	80%<br>90%	1070	1000	70	-0.135<br>-0.161	0.85<br>0.85	0.72<br>0.69
0.05	80%<br>90%	1070	1000	70	-0.149<br>-0.175	0.80<br>0.80	0.65<br>0.63

Hypothesis 6:

Comparisons between ITIP3 and ITIP2

The percent of children who are cured by day 14 will be lower among children who have HIV and chest-indrawing pneumonia who are excluded from the trials than among children in the standard care arm in the ITIP2 trial.

We present estimates of effect size (column “delta”) (difference in percent of children who are cured by day 14 under the assumption that the ITIP3 will enroll 1000 children and that either 5% or 10% have HIV with a two-sided test of proportions, an alpha level of 0.05 and 80%, 85% or 90% power.

Prevalence of HIV of 5% and percent of outcome of 90%, 85% and 80% in the ITIP2 (referent) group:

alpha	Power	N	N1	N2	delta	p1	p2
0.05	80%<br>90%	1050	1000	50	-0.139<br>-0.170	0.90<br>0.90	0.76<br>0.73

0.05	80%<br>90%	1050	1000	50	-0.159<br>-0.191	0.85<br>0.85	0.69<br>0.66
0.05	80%<br>90%	1050	1000	50	-0.174<br>-0.206	0.80<br>0.80	0.63<br>0.59

Prevalence of HIV of 10% and percent of outcome of 90%, 85% and 80% (column p1) in the ITIP2 (referent) group:

**alpha**	**Power**	**N**	**N1**	**N2**	**delta**	**p1**	**p2**
0.05	80%<br>90%	1100	1000	100	-0.099<br>-0.119	0.90<br>0.90	0.80<br>0.78
0.05	80%<br>90%	1100	1000	100	-0.114<br>-0.136	0.85<br>0.85	0.74<br>0.71
0.05	80%<br>90%	1100	1000	100	-0.125<br>-0.147	0.80<br>0.80	0.68<br>0.65

APPENDIX IV: STUDY REQUIREMENTS AND TRAINING

Additional study requirements not already described in the protocol are summarized below.

- **Personnel**

  The study team on-the-ground will consist of full-time employees in the following capacities:

  - Study coordinator: clinician who will oversee daily study operations and monitor the safety of participants
  - Pharmacists: receive, account for, prepare, and distribute study product; train study staff on study product administration procedures
  - Data manager: maintain study databases with quality control and quality assurance procedures; prepare regular reports of ongoing study activities and data
  - Data clerk/administrator: scan data collected on paper forms for entry into electronic database(s); maintain copies of study documents as needed
  - Study nurses: perform screening, enrollment, follow-up and interim study visit procedures; conduct informed consent process; conduct all home visits and study retention efforts.
  - HTC counselors: perform HIV test pre-counseling, testing and post-test counseling per study protocol and Malawi national guidelines.
  - Fieldworkers: perform home follow up visits.

  In addition to the full-time staff, the project will use the expertise of additional SCUS/SCI and UNC personnel to assist with meeting study goals. A portion of SCUS/SCI and UNC staff's time will be for operational and grant management services, which will provide a range of support activities to the project such as accounting, human resources management, information technology, administration and audit services.

  Government staff at the study site hospital will also be engaged with this study. KCH or BDH service providers will be responsible for recruiting of study participants and managing participants' care while in the hospital.

- **Training**

  All study staff will be trained in the Protection of Human Subjects prior to any interactions with study participants. Additionally, before the study starts, all study staff will attend an extensive 5-day study-specific training to review all study procedures, including the study protocol, SOPs, data collection tools, informed consent process, reporting requirements, and safety monitoring. Refresher trainings on the identification of pneumonia will be scheduled at least once per year and will include updates from the study monitor reports. Trainings will be conducted by a Sponsor representative,

representative of the study CRO, or other qualified clinician, as appropriate for the training material.

Government staff at KCH and BDH will be sensitized to this study and will receive at least one day of training on the identification of pneumonia and study-specific procedures and documentation prior to the study start. Refresher trainings will be held periodically, at least once every year.

- **Supplies**

  Supplies for this study include the following:
  - Laptops for study staff
  - Printer
  - Photocopier
  - Respiratory rate counters
  - Portable pulse oximeter
  - Scale
  - Height board
  - Malaria RDT kits
  - Office furniture
  - Partitions/privacy screens for the study clinic
  - Communication equipment such as cellphone accessories, airtime, and internet sticks
  - Standard office supplies, including binders, paper, pens

- **Transportation**

  The study will obtain multiple motorcycles for use by the study retention team to conduct home visits after a participant misses a scheduled study visit. Study participants will be expected to provide their own transportation to study visits at KCH, but will receive a travel reimbursement.

- **Space**

  The study clinic for out-patient screening, enrollment, follow-up and interim visits will be located in the OPD of KCH or BDH and wards of KCH. The hospital has provided the study with a private room for study visits and other study-related activities. Additional office space for data management and the study coordinator will be provided at a separate location in Lilongwe.

APPENDIX V: STUDY SENSITIZATION/RECRUITMENT SCRIPT

Instructions: This script is to be used by Kamuzu Central Hospital and Bwaila District Hospital staff in the Paediatric Outpatient Department after the initial triage and intake of a presenting child. This content should be presented to caregivers of children who are between 2 and 59 months of age and have cough or difficult breathing.

Script: "There are three ongoing research studies for children with pneumonia and your child may be eligible to participate in one of them. The studies are investigating different treatment regimens for childhood pneumonia, seeing if less antibiotic use is as effective for curing pneumonia. If you are interested in learning more about these studies, I can let the study staff know that it is okay to contact you. If you aren't interested in the studies, that is fine and no one from the study will contact you about them. Your decision to participate in a study will not affect the medical care that your child receives in the hospital. Are you interested in learning more about the studies?"

Prompts: If families ask other questions about the study, including procedures, risks, or benefits, they will be referred to study staff.
