# Supplementary material for: Clinical Outcomes of Pneumonia and Other Comorbidities in Children Aged 2-59 Months in Lilongwe, Malawi: Protocol for the Prospective Observational Study “Innovative Treatments in Pneumonia”
Source: JMIR Res Protoc. 2019 Jul 29;8(7):e13377. doi: 10.2196/13377 (PMC6690162; doi:10.2196/13377)
Supplement: Multimedia Appendix 2 [file resprot_v8i7e13377_app2.pdf]

Appendix II: ITIP1 (fast breathing pneumonia clinical trial) follow-up visit schedule

	Day 1	Day 2	Day 3	Day 4	Day 14
**Study activities**	• Enrollment visit	• Follow-up visit	• Follow-up visit	• Follow-up visit	• Follow-up visit and study exit

Appendix III: ITIP2 (chest indrawing pneumonia clinical trial) follow-up visit schedule

	Day 1	Day 2	Day 4	Day 6	Day 14
**Study activities**	• Enrollment visit	• Follow-up visit	• Follow-up visit	• Follow-up visit	• Follow-up visit and study exit
